# Highly distinct chromosomal structures in cowpea (*Vigna unguiculata*), as revealed by molecular cytogenetic analysis

**DOI:** 10.1007/s10577-015-9515-3

**Published:** 2016-01-12

**Authors:** Aiko Iwata-Otsubo, Jer-Young Lin, Navdeep Gill, Scott A. Jackson

**Affiliations:** Center for Applied Genetic Technologies, University of Georgia, 111 Riverbend Road, Athens, GA 30602 USA; Department of Agronomy, Purdue University, 170 S. University Street, West Lafayette, IN USA; Department of Biology, University of Pennsylvania, Philadelphia, 19104 PA USA; Department of Molecular, Cell, and Developmental Biology, University of California, Los Angeles, CA 90095 USA; Department of Botany, University of British Columbia, Vancouver, British Columbia V6T 1Z4 Canada

**Keywords:** *Vigna unguiculata*, Molecular cytogenetics, Retrotransposon, Centromere

## Abstract

Cowpea (*Vigna unguiculata* (L.) Walp) is an important legume, particularly in developing countries. However, little is known about its genome or chromosome structure. We used molecular cytogenetics to characterize the structure of pachytene chromosomes to advance our knowledge of chromosome and genome organization of cowpea. Our data showed that cowpea has highly distinct chromosomal structures that are cytologically visible as brightly DAPI-stained heterochromatic regions. Analysis of the repetitive fraction of the cowpea genome present at centromeric and pericentromeric regions confirmed that two retrotransposons are major components of pericentromeric regions and that a 455-bp tandem repeat is found at seven out of 11 centromere pairs in cowpea. These repeats likely evolved after the divergence of cowpea from common bean and form chromosomal structure unique to cowpea. The integration of cowpea genetic and physical chromosome maps reveals potential regions of suppressed recombination due to condensed heterochromatin and a lack of pairing in a few chromosomal termini. This study provides fundamental knowledge on cowpea chromosome structure and molecular cytogenetics tools for further chromosome studies.

**Electronic supplementary material:**

The online version of this article (doi:10.1007/s10577-015-9515-3) contains supplementary material, which is available to authorized users.

## Introduction

Cowpea (*Vigna unguiculata* (L.) Walp) is an important agronomic crop in Africa and other developing countries and is particularly tolerant to drought and heat stress, especially as compared to other legume crops (Hall 2004). Cowpea is closely related to other economically important legumes such as soybean (*Glycine max*), common bean (*Phaseolus vulgaris*), and pigeonpea (*Cajanus cajan*) and has a relatively small genome size of 620 Mb, consisting of 2*n* = 2*x* = 22 chromosomes (Arumuganathan and Earle 1991). Cowpea was identified as an “orphan crop” with limited genomic resources (Naylor et al. 2004); however, since then, a consensus genetic map with high-density single-nucleotide polymorphism (SNP) markers has been developed from 11 mapping populations (Muchero et al. 2009; Lucas et al. 2011; consensus genetic linkage map version 6 in http://harvest.ucr.edu). Extensive genomic resources such as bacterial artificial chromosome (BAC) libraries, BAC end sequences (BESs), and a physical map have been constructed. Moreover, there is an ongoing genome sequencing project (https://www.integratedbreeding.net/126/communities/genomics-crop-info/agricultural-genomics/genome-sequencing/cowpea). These resources have and will facilitate marker-assisted breeding, association mapping, and comparative analyses of cowpea with other crops. In addition, whole genome sequencing projects on other important *Vigna* crops such as mung bean (*Vigna radiata*) and azuki bean (*Vigna angularis*) have been published (Kang et al. 2014, 2015), which will accelerate the development of genetic and genomic resources for these closely related species and allow them to leverage each other to advance our ability to genetically manipulate these crops.

Despite recent rapid advances in the establishment of genetic and genomic resources, association between genomic information and chromosomal organization of cowpea has been limited. A cowpea karyotype, chromosomal banding patterns, karyotype comparisons among wild cowpea species, chromosomal distribution of ribosomal DNA (rDNA), a centromeric repetitive DNA family, and Ty1-copia-like retrotransposable elements have been previously reported (Barone and Saccardo 1990; Pignone et al. 1990; Saccardo et al. 1992; Galasso et al. 1995, 1997; Guerra et al. 1996; Venora and Padulosi 1997). A comparative cytogenetics study between cowpea and common bean was done using common bean BAC clones mapped to cowpea mitotic chromosomes (Vasconcelos et al. 2015). Polytene chromosomes from anther tapetum cells of *Vigna* species have been observed and used for cytogenetic studies (reviewed in Guerra 2001). However, most of these studies were done when genomic resources were limited and do not provide a detailed genome organization associated with chromosome structures.

With the development of BAC libraries, fluorescence in situ hybridization (FISH) using BAC clones as probes became a powerful tool in modern cytogenetics. FISH using genetically anchored single- or low-copy BAC probes has been used to integrate genetic and chromosome maps in numerous plant species. This approach has helped to identify individual chromosomes, reveal recombination patterns, find discrepancies between genetic and chromosome maps, and correlate genetic markers with cytological features such as telomeres, heterochromatin, and euchromatin (Cheng et al. 2001a, b; Kulikova et al. 2001; Zhang et al. 2005; Wang et al. 2006a; Fonseca et al. 2010; Ohmido et al. 2010; Iovene et al. 2011).

FISH using repetitive BAC probes has been used to determine chromosomal distribution of the repeats (Lin et al. 2005; Fonseca et al. 2010; Xiong and Pires 2011). In higher plant species, repetitive DNA sequences represent a large fraction of most genomes. For example, in soybean, ∼59 % of the genome is made up of repetitive elements (Schmutz et al. 2010). Based on chromosomal organization in other plants, repetitive sequences are typically enriched in the pericentromeric heterochromatic regions. The pericentromeric regions of soybean contain fast-evolving tandem repeats with interspersed retroelements (Lin et al. 2005). Thus far, in cowpea, only Ty1-copia-type retrotransposons have been identified, which were dispersed relatively uniformly across all chromosomes except in centromeres and subtelomeres (Galasso et al. 1997).

Centromeric regions in plants are typically rich in satellite repeats (a type of tandem repeat) and retrotransposons (reviewed in Jiang et al. 2003). Despite the functional conservation of centromeres, centromeric satellite repeats across species are highly diverged. Of the identified satellite repeats in plant species, for example, CentO in rice, CentC in maize, pAL in *Arabidopsis*, CentGm in soybean, and CentPv in common bean, all have unit sizes that range between ∼90 and ∼180 bp (Martinez-Zapater et al. 1986; Murata et al. 1994; Ananiev et al. 1998; Cheng et al. 2002; Gill et al. 2009; Iwata et al. 2013). In cowpea, a 488-bp AT-rich centromeric repetitive sequence isolated from a *Dra*I digestion of genomic DNA was previously reported (Galasso et al. 1995). However, it is unknown if this 488-bp repeat is part of satellite repeats or derived from some type of transposable element, and what other repetitive components compose centromeres in cowpea. In common bean, which diverged from cowpea ∼5 million years ago (MYA), two centromeric satellite repeats, CentPv1 and CentPv2, were identified (Iwata et al. 2013). Southern analysis showed that neither of these repeats was conserved in cowpea, indicating that other satellite repeats might dominate cowpea centromeres.

In this study, we utilized available genomic resources with the aim of advancing our knowledge of chromosome organization in cowpea. Molecular cytogenetics analysis and integration of genetic and physical chromosome maps revealed highly distinct structure of cowpea chromosomes. This study contributes to a better understanding of chromosome organization of cowpea and facilitates an understanding of chromosome/genome evolution in legumes.

## Materials and methods

### Plant materials

Blackeye 5 line 9405C and African accession IT97K-499-35 were grown in greenhouse and field for DNA extraction and preparations of mitotic and meiotic chromosomes.

### Preparation of mitotic and meiotic chromosomes

Mitotic chromosome preparations were conducted as previously described (Gill et al. 2009 and Findley et al. 2010) with the following modifications. Root tips from potted plants were treated with pressurized nitrous oxide for 90 min, fixed in a solution composed of 3:1 ethanol and glacial acetic acid for a day at room temperature, and then stored at 4 °C. After rinsing fixed root tips in distilled water, root tips were digested with an enzyme solution containing 1 % Pectolyase (MP Biomedicals) and 2 % Cellulase (MP Biomedicals) in citric buffer (10 mM sodium citrate, 10 mM sodium EDTA, pH 5.5) for 80 min at 37 ° C. For meiotic chromosome preparation, tiny flower buds (1∼2 mm) of cowpea grown in field were collected and fixed in 3:1 ethanol and glacial acetic acid for 24 h at room temperature and then stored at 4 °C. Flower buds were dissected under dissecting microscope and anthers around 0.6 mm in length were selected for preparation of meiotic pachytene chromosome spreads. The preparation was based on the published protocol with the following modifications (Ross et al. 1996). The selected anthers were incubated in an enzyme mixture containing 1 % (*w*/*v*) Pectolyase (MP Biomedicals) and 2 % (*w*/*v*) Cellulase (MP Biomedicals) in citric buffer (10 mM sodium citrate, 10 mM sodium EDTA, pH 5.5) for 2.5 h at 37 °C. The digested anthers were macerated on glass slides in 60 % acetic acids at 50 °C with fine forceps. Subsequently, ice-cold 3:1 ethanol and glacial acetic acid were added to the slide, and the slide was dried.

### Identification of highly abundant tandem repeats

To identify potential centromeric satellite repeats, we searched for highly abundant tandem repeats in the cowpea genome. First, tandem repeats were searched against the BAC end sequences (BESs) of VUH2 BAC library (NCBI GI 146506166-146551943 and GI 270244997-270250498) using tandem repeats finder (TRF; Benson 1999), and tandem repeats with a consensus size greater than 60 bp were further analyzed. In order to find highly abundant tandem repeats in the whole genome, we used a randomly selected subset of 26-bp (26mer) sequences of top 10,000 highest copy numbers corresponding to the 26-bp sequences occurring more than 271,512 times in a 60× draft genome. We performed BLAST analysis using the subset of 26-bp sequences as queries against the BES-derived tandem repeats.

### Fluorescence in situ hybridization

BAC clones anchored to linkage groups were selected and used as a FISH probe (Table 1; Muchero et al. 2009). BAC DNAs were extracted using QIAGEN large construct kit. The 455- and 285-bp tandem repeats were amplified from genomic DNA of IT97K-499-35 using primer sets P1 and P2, respectively (Supplemental Table 1). The retrotransposon gag-pol region of VUH2_70J18 and the retrotransposon long terminal repeat (LTR) region of VUH2_81M23 were amplified from each BAC clone using primer sets of P3 and P4, respectively (Supplemental Table 1). The amplified PCR fragments were purified from agarose gel and used for a FISH probe. An 18S rDNA clone developed from soybean was provided by D.A. Johnson at University of Ottawa, and 5S rDNA clone was cloned from common bean. FISH was carried out according to Walling et al. 2005. BAC DNAs, rDNAs, and the purified PCR products were nick translated with either biotin dUTP or digoxigenin dUTP (Roche) and visualized with Streptavidin Alexa Fluor 488 (Invitrogen) or Anti-Digoxigenin-Rhodamine (Roche), respectively. FISH with 176-bp tandem repeat and 18S rDNA was conducted using an oligonucleotide probes (5′-AATACCATGAAAGTCTTGGTGCAC-3′) labeled with FAM (Integrated DNA Technologies) and 18S rDNA directly labeled with Cy5, according to the published protocol (Gill et al. 2009; Iwata et al. 2013). The images were taken with Zeiss Axio Imager M2 microscope, equipped with AxioCam MRm, controlled by Axio Vision 40 V4.8.2.0. The images were adjusted for publication using Adobe Photoshop CS5.1 (Adobe Systems Incorporated). The chromosome lengths were measured using Axio Vision 40. V4.8.2.0. Straightened chromosome images were obtained using ImageJ (Kocsis et al. 1991). Pachytene chromosomes were numbered according to their corresponding linkage groups (Muchero et al. 2009).

**Table 1:** Genetic and physical positions of the cowpea BAC clones and rDNA on pachytene chromosomes

Linkage group	Entire linkage group length (cM)	BAC clone	SNP markers	Genetic position (cM)^a^	Relative genetic position^b^	Physical location^c^	*n*
1	59	H014O11	1_0731	–	–	3.24 ± 1.68	16
		M002E09	1_1278	3.3	5.6	24.62 ± 2.37	19
			1_0012	–	–		
		H061J06	1_1193	48.4	82	65.16 ± 3.13	19
2	71.6	H088A15	1_1067	5.2	7.3	3.53 ± 0.59	23
			1_0852	–	–		
		H036P04	1_1495	69.5	97.1	96.9 ± 0.58	23
			1_1527	69.5	97.1		
3	105.4	H037B01	1_0852	–	–	1.02 ± 0.31	23
			1_1143	–	–		
			1_0447	2.2	2.1		
			1_0822	–	–		
		M045O05	1_0380	57.7	54.7	53.34 ± 2.81	23
			1_0984	57.5	54.6		
4	45.3	M062M10	1_0973	1.9	4.2	7.3 ± 1.10	15
		H031B04	1_1146	44.5	98.2	99.85 ± 0.39	18
			1_0122	44.5	98.2		
			1_0267	44.5	98.2		
5	63.1	M057N05	1_0409	–	–	0 ± 0	10
			1_0684	0	0		
		H039A20	1_0387	59.4	94.1	94.76 ± 1.19	12
			1_0466	–	–		
			1_0935	58.9	93.3		
			1_0579	59.2	93.8		
6	59.9	H065G04	1_0024	6.9	11.5	93.37 ± 2.04	18
			1_0823	–	–		
7	50.1	H074C16	1_1141	10.9	21.8	30.37 ± 4.23	16
			1_0198	10.9	21.8		
		M026L23	1_0559	39.2	78.2	82.41 ± 2.03	16
8	60.4	H086N19	1_0558	11.5	19	15.35 ± 1.22	16
		H010M18	1_1503	–	–	98.02 ± 0.35	16
			1_0058	57.7	95.5		
			1_1529	58	96		
9	42.8	M054N15	1_0257	36.9	86.2	11.66 ± 1.84	19
			1_1255	36.9	86.2		
		5S rDNA		–	–	25.32 ± 2.53	13
		M045J08	1_0651	9	21	83.39 ± 2.39	19
10	63.2	H025N06	1_0282	61.3	97	4.39 ± 0.72	14
		5S rDNA		–	–	56.69 ± 3.14	16
		H015M15	1_1120	0.3	0.5	98.96 ± 0.28	14
			1_0977	0.3	0.5		
			1_1407	0	0		
			1_1089	0	0		
11	59.3	H049E24	1_1269	–	–	0 ± 0	13
			1_1308	–	–		
			1_0940	0	0		
		H085I15	1_1493	–	–	88.36 ± 1.64	13
			1_0606	43.6	73.5		

*n* Number of measurements

^a^Genetic position is based on cowpea genetic map v6 (http://harvest.ucr.edu/)

^b^Relative genetic position is calculated using the following formula: the cM value of SNP marker on the genetic map / the total cM values of the same linkage map ∗ 100

^c^Physical location is calculated using the following formula: the distance (in μm) from the FISH signal to the end of the short arm of the chromosome / the total length (in μm) of the chromosome. The short and long arms are defined according to the linkage map orientation

### Bacterial artificial chromosome DNA sequencing and analysis

BAC DNAs of VUH2_81M23 (NCBI ID: 24466881) and VUH2_70J18 (NCBI ID: 24462974) were extracted using QIAGEN large construct kit (BAC 70J18 subsequent to sequencing was lost due to library contamination; however, sequences are available from the original colony described here). For sequencing of VUH2_81M23, shotgun clone library for BAC sequencing was constructed as previously described in SanMiguel et al. (2002) and Lin et al. (2005). VUH2_81M23 was sequenced from both directions using Big Dye Terminator chemistry (Applied Biosystems) and run on an ABI3730 sequencer. Base calling and quality assessment were done using PHRED, assembled by PHRAP, and edited with CONSED (Ewing et al. 1998; Gordon et al. 1998). FGENESH (www.softberry.com/) was used for de novo gene prediction. Predicted genes were annotated using deduced amino acid sequences by BLASTP against the NCBI nonredundant protein database with an e-value cutoff of 10^−10^. Predicted coding segments (CDS) were used for annotating genes using BLASTX against the NCBI nonredundant protein database with an e-value of 10^−10^. Repetitive sequences were annotated using de novo methods and homology searches. De novo identification of LTR retrotransposons was done using LTR-struc (McCarthy and McDonald 2003) and LTR-finder (Xu and Wang 2007). Homology searches for LTR retrotransposons and DNA transposons were conducted using BLASTX of FGENESH predicted gene models against the NCBI nonredundant protein database. An exhaustive search for repetitive sequence was performed using RepeatMasker (www.repeatmasker.org/) with a customized cowpea repeat database created by RECON analysis (Bao and Eddy 2002) from VUH2 BESs.

The DNA of VUH2_70J18 was sequenced using Roche 454 sequencing at the University of Georgia. With a mean size of 515 bp, 160,945 reads were obtained of which 91,460 nonredundant reads were used for repeat analysis. Similarity-based clustering of the reads, assembly of the reads, and classification of repeats in individual clusters were performed using RepeatExplorer (Novak et al. 2013).

### Immunodetection of 5-methylcytosine

Postfixation of the slides was performed according to the published protocol (Lysak et al. 2006). Briefly, the slides were postfixed in 4 % formaldehyde in 1× phosphate-buffered saline (PBS) for 10 min at room temperature, washed twice in 1× PBS for 5 min each, and dehydrated in an ethanol series (70, 90, and 100 %). The immunodetection was performed according to Zhang et al. (2008) using mouse anti-5-methylcytosine (1:500, Eurogentec) detected with Alexa Flour 568 goat anti-mouse IgG (Life Technologies). The chromosomes were counterstained with 4′,6-diamidino-2-phenylindole (DAPI). Images were taken with Zeiss Axio Imager M2 microscope, equipped with AxioCam MRm, and controlled by Axio Vision 40 V4.8.2.0. Adobe Photoshop CS5 (Adobe Systems Incorporated) was used to produce publication images.

## Results

### Microscopic analysis of cowpea chromosomal structure

**Fig. 1:**
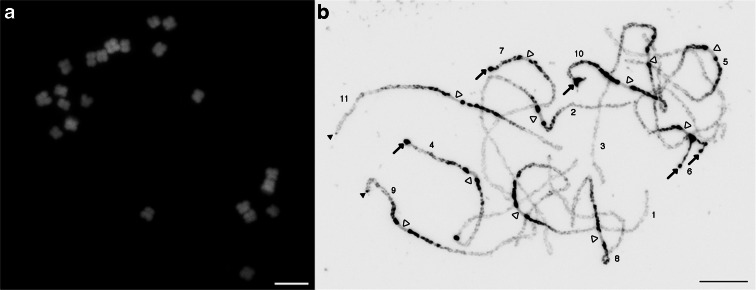
Cowpea chromosomes stained with 4′,6-diamidino-2-phenylindole (DAPI). **a** Mitotic metaphase chromosomes. *Bar* = 5 μm. **b** Meiotic pachytene chromosomes. The image was converted into *black* and *white* to enhance the visualization of cytological features along the pachytene chromosomes. Chromosomes were distinguished and identified based on centromeric positions, heterochromatin distributions, and chromosome lengths and numbered according to the corresponding linkage groups. *Opened arrowheads* indicate the centromere positions based on the chromatin structure. *Bar* = 10 μm. *Arrows* show large heterochromatic knobs at chromosomal termini of 4S, 6S, 7S, and 10S. There are two heterochromatic knobs at chromosome 6S due to unpaired chromosomal termini. *Closed arrowheads* show small heterochromatic knobs at chromosomal termini of 9S and 11L

We first analyzed the chromosomal structure of cowpea using DAPI staining (Fig. 1). Consistent with previous reports (Galasso et al. 1995; Vasconcelos et al. 2015), cowpea mitotic metaphase chromosomes were small (2–3 μm) and most were metacentric or submetacentric (Fig. 1a). The individual chromosomes were morphologically similar, and it was nearly impossible to distinguish homologous chromosomes pairs.

**Fig. 2:**
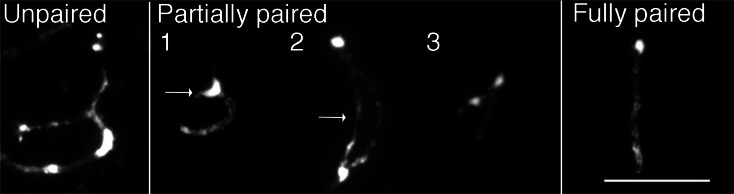
Five different chromosomal termini are categorized into three types of structures at chromosomal termini with big heterochromatic knobs. Only chromosomal termini were cropped and shown in the figure. *Unpaired*: euchromatic subtelomeric regions and heterochromatic knobs are completely unpaired. *Partially paired*: (1) euchromatic subtelomeric regions are paired, and heterochromatic knobs are somewhat paired, but not completely, showing a heart-like structure (*arrow*); (2) euchromatic subtelomeric regions are unpaired, but heterochromatic knobs are paired showing a bubble-shape structure (*arrow*); and (3) euchromatic subtelomeric regions are paired, but heterochromatic knobs are unpaired. *Fully paired*: euchromatic subtelomeric regions and heterochromatic knobs are completely paired. *Bar* = 10 μm

We next analyzed meiotic pachytene chromosomes derived from cowpea pollen mother cells (Fig. 1b). Pachytene chromosomes are often used for cytological studies in plant species, as they show conspicuous cytological features. As seen in other plants such as rice and *Medicago* (Cheng et al. 2001b; Kulikova et al. 2001), pericentromeric regions of all chromosomes were composed of highly condensed heterochromatin blocks with the chromosome arms being mostly euchromatic (Fig. 1b). There were several interesting cytological features unique to cowpea pachytene chromosomes, not even observed in other closely related legume species such as soybean and common bean (Walling et al. 2006; David et al. 2009). First, centromeres were cytologically larger (∼2.7 μm on average) as compared to other plant species (Cheng et al. 2001b; Zhang et al. 2005; Tang et al. 2009; Iovene et al. 2011), and based on DAPI staining, the centromeric chromatin looked markedly different from heterochromatic and euchromatic regions (open arrowheads in Fig. 1b). Second, some of the heterochromatin was highly distinct to form “knob-like” structures, particularly around the pericentromeres and telomeres. Pericentromeric heterochromatic knobs were found flanking all 11 centromeres. Four chromosomal termini had large heterochromatic knob structures (arrows in Fig. 1b); these regions were found to correspond to chromosomes 4S, 6S, 7S, and 10S. Two chromosomal termini (9S and 11L) possessed small knobs (filled arrowheads in Fig. 1b). Third, the chromosomal termini with large heterochromatin have euchromatic subtelomeric regions where homologous chromosomes were not always completely paired—chromosomes 4S, 6S, 7S, and 10S. These unpaired chromosomal termini were found in early to late pachytene stages. Based on the analysis of 50 pollen mother cells, the structure of these four chromosomal termini was grouped into three types: unpaired, partially paired, and fully paired (Fig. 2; Supplemental Table 2).

### Fluorescence in situ hybridization analysis of ribosomal DNA

To further investigate chromosomal organization related to pachytene chromosome structure, 18S and 5S rDNAs were mapped to pachytene chromosomes using FISH (Supplemental Fig. 1). Strong 18S rDNA signals coincided with the large heterochromatin knobs at the four chromosomal termini (chromosome 4S, 6S, 7S, and 10S). One additional interstitial locus was detected in the middle of a chromosome (arrow in Supplemental Fig. 1; later determined to be chromosome 8S by FISH; unpublished data). Weak 18S rDNA signals were also detected at a small heterochromatic knob on chromosome 9S (Supplemental Fig. 1, arrowhead). Therefore, we concluded that 18S rDNA is the major component of heterochromatic knobs at chromosomal termini. Two 5S rDNA loci were found. One pair of chromosomes possessed both 18S rDNA and 5S rDNA on opposite arms, which is homologous to chromosome 10 of common bean (Vasconcelos et al. 2015).

### Integration of linkage groups with the chromosome map

**Fig. 3:**
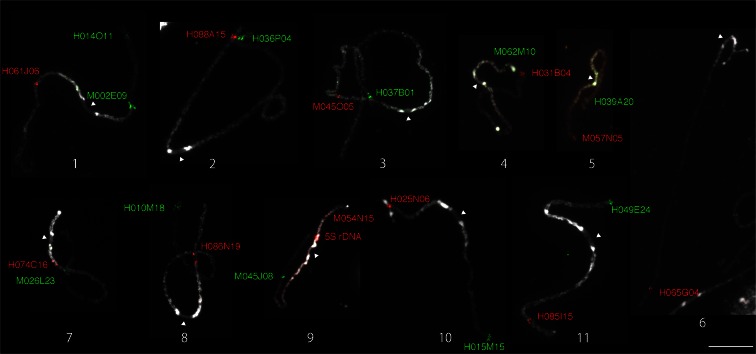
Cowpea pachytene chromosomes probed with chromosome-specific BAC clones (listed in Table 1). *Arrowheads* indicate centromeric positions based on chromatin staining. *Bar* = 10 μm. The names of BAC clones mapped to individual chromosomes using FISH are shown next to signals in *green* or *red* corresponding to their signal *colors*

**Fig. 4:**
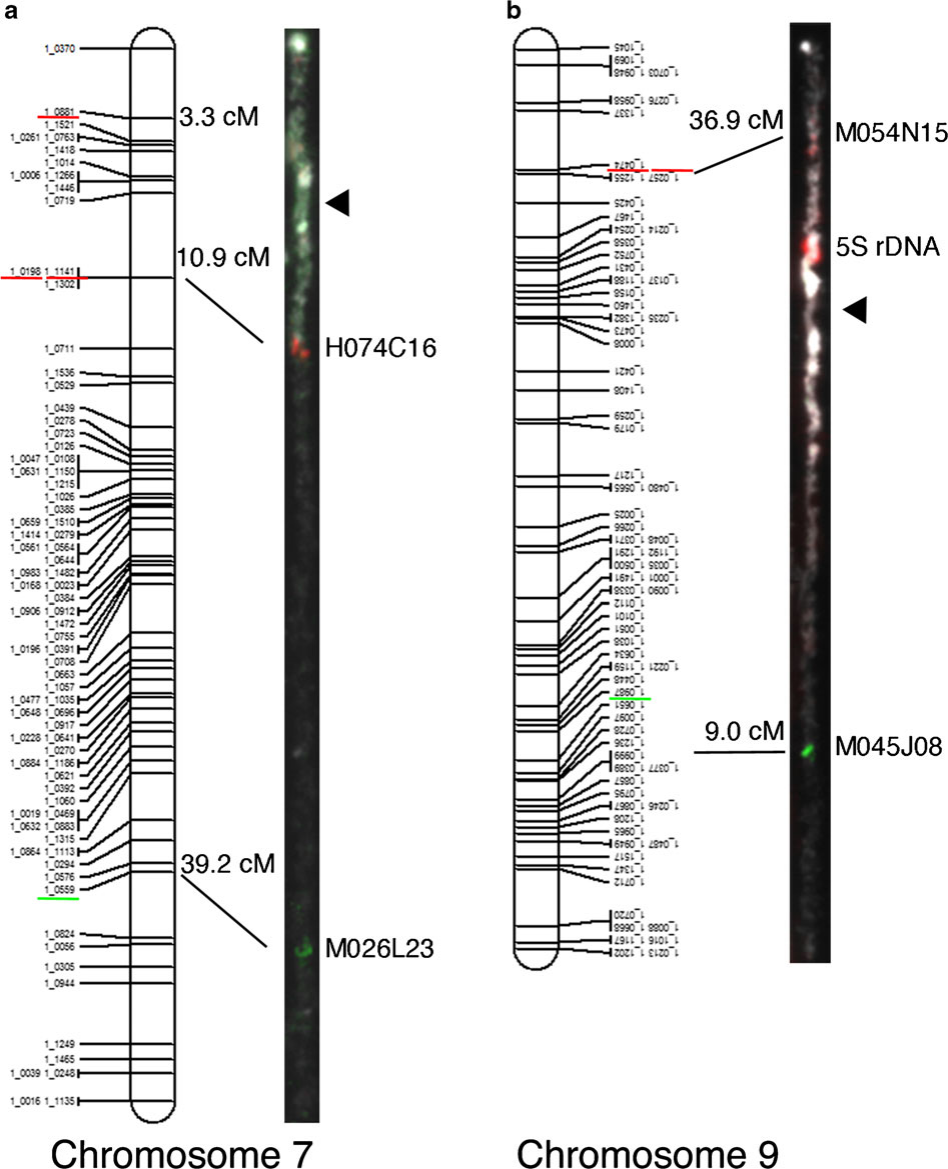
Comparisons between linkage (Muchero et al. 2009) and cytogenetic maps. FISH images of pachytene chromosomes straightened and lengths adjusted to those of linkage map for relative distance comparisons. Centromeres on chromosomes are indicated by *arrowheads*. **a** Chromosome 7. **b** Chromosome 9

Mapping of genetically anchored cloned sequences to pachytene chromosomes has been used to investigate the position of individual genetic markers on chromosomes, identify recombination hot/cold spots, evaluate the accuracy of linkage mapping, and associate chromosomal structures with genetic markers (Cheng et al. 2001a; Kulikova et al. 2001; Zhang et al. 2005; Wang et al. 2006a; Szinay et al. 2008; Ohmido et al. 2010; Iovene et al. 2011). Twenty-two cowpea BAC clones were selected and mapped to individual chromosomes using FISH (Table 1; Fig. 3). Most BAC clones were genetically anchored near the ends of linkage groups, and as expected, their signals localized to subtelomeric regions (Table 1). The exception was H074C16, which was genetically anchored to the short arm of linkage group 7; however, the FISH signal was detected close to centromere and on the same arm as M026L23, which was genetically anchored to long arm of linkage group 7 (Fig. 4a). To obtain a better FISH marker for the short arm of chromosome 7, M051D16, anchored to short arm of linkage group 7 (3.3 cM), was also tested, but the FISH signal also localized on the long arm (Supplemental Fig. 2). This suggests that there are no or very few genetic markers on the short arm of chromosome 7.

Another discrepancy observed between genetic and chromosome maps was the orientation of genetic maps for chromosomes 6, 9, and 10 (example of chromosome 9 in Fig. 4b). For consistency, we refer to the cytologically defined long and short arms; thus, the genetic maps for chromosomes 6, 9, and 10 are inverted relative to the chromosome orientation.

### Identification and characterization of individual pachytene chromosomes

**Fig. 5:**
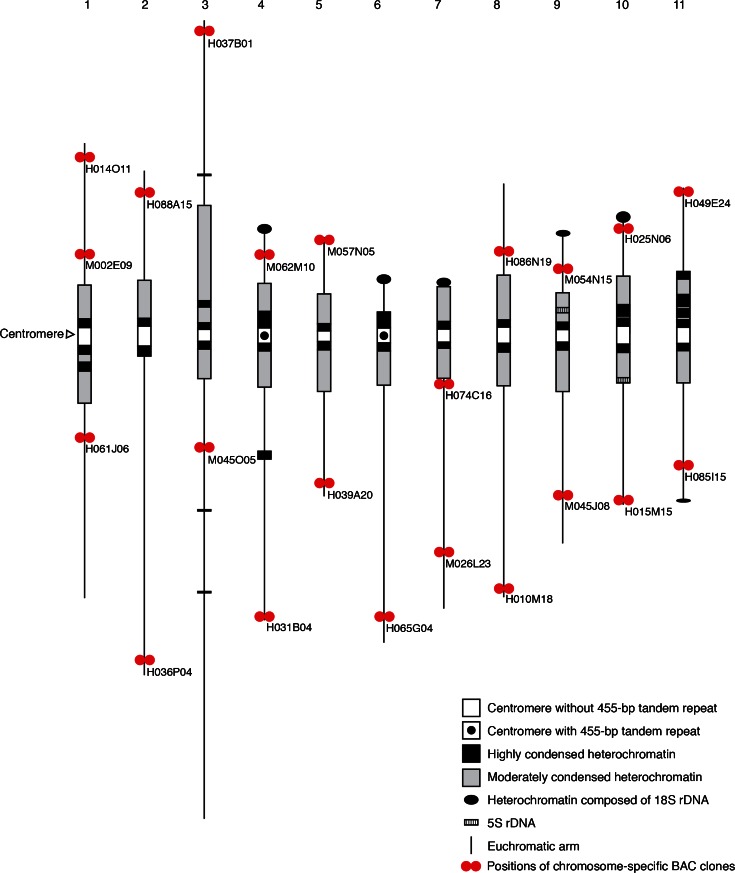
Ideogram of 11 pachytene chromosomes of cowpea with heterochromatin distribution, centromere position, chromosome lengths, and positions of 18S rDNA, 5S rDNA, and 455-bp tandem repeat

Cowpea pachytene chromosomes are relatively long and were often entangled; thus, in a single cell, it was difficult to trace all of 11 chromosomes from end to end. Therefore, different cells were used to collect the 11 chromosome images used to measure chromosome lengths and analyze cytological features (Table 2). Chromosome lengths averaged 67.72 μm with a range of ∼127.06 μm (chromosome 3) to 45.66 μm (chromosome 5). Chromosome identification was done using chromosome-specific BAC clones for FISH, but it was also possible to distinguish individual chromosomes by cytological features including heterochromatin distribution, chromosome lengths, and arm ratios. We developed ideogram showing cytological features and BAC positions (Fig. 5).

**Table 2:** Karyotype of cowpea pachytene chromosomes

Chromosome number^a^	1	2	3	4	5	6	7	8	9	10	11
Short arm^b^ (μm)	34.04 ± 7.02	27.52 ± 5.24	50.05 ± 10.50	16.84 ± 3.16	16.09 ± 3.59	9.65 ± 1.61	9.59 ± 2.55	25.86 ± 3.46	18.4 ± 2.97	21.26 ± 5.24	23.54 ± 4.78
Long arm (μm)	45.39 ± 11.27	57.90 ± 9.94	77.01 ± 17.07	45.67 ± 10.40	27.63 ± 5.83	49.01 ± 12.6	47.45 ± 12.27	46.35 ± 7.58	36.18 ± 7.55	29.15 ± 7.36	28.38 ± 5.99
Total length (μm)	79.43 ± 17.89	85.42 ± 14.11	127.06 ± 26.95	62.51 ± 12.60	45.66 ± 9.03	58.64 ± 13.26	57.04 ± 14.36	72.2 ± 10.14	54.59 ± 9.84	50.41 ± 21.93	51.92 ± 10.65
Relative chromosome length (%)^c^	10.66	11.47	17.06	8.39	6.13	7.87	7.66	9.69	7.33	6.77	6.97
Arm ratio (L/S)	1.33 ± 0.14	2.13 ± 0.35	1.54 ± 0.16	2.73 ± 0.49	1.65 ± 0.19	5.14 ± 1.3	5.02 ± 0.93	1.80 ± 0.24	1.97 ± 0.29	1.38 ± 0.17	1.27 ± 0.05
*n*	20	23	25	25	16	28	24	16	32	30	16
Heterochromatic knob at chromosomal termini	–	–	–	S, Large	–	S, Large	S, Large	–	S, Small	S, Large	L, Small
18S rDNA^d^	–	–	–	S	–	S	S	–	S	S	–
5S rDNA	–	–	–	–	–	–	–	–	S	L	–
Heterochromatic knob in euchromatic arm	–	–	–	L	–	–	–	–	–	–	–
455-bp tandem repeats	Cen	Cen	–	Cen	Cen	Cen	–	–	–	Cen	Cen

*n* The number of measurements, *S* short arm, *L* long arm, *Cen* centromere

^a^Chromosomes are numbered according to their linkage groups (Muchero et al. 2009)

^b^Short and long arms are defined according to actual lengths of individual pachytene chromosomes

^c^Relative chromosome length is a percentage of chromosome length/total length of all chromosomes

^d^A chromosome that has 18S rDNA at pericentromeric heterochromatin is not identified

### Molecular characterization of pericentromeric heterochromatin

Microscopic observation of pachytene chromosomes revealed heterochromatin in pericentromeric regions, indicating the presence of repetitive sequences in these regions. It was shown that repetitive sequences at pericentromeric regions of soybean were not conserved in *Vigna* species (Lin et al. 2005). To analyze the genome organization of cowpea pericentromeric regions, we used two BAC clones selected randomly from a BAC library of VUH2 Blackeye 5 line 9405C, VUH2_70J18 and VUH2_81M23, both containing repetitive sequences.

**Fig. 6:**
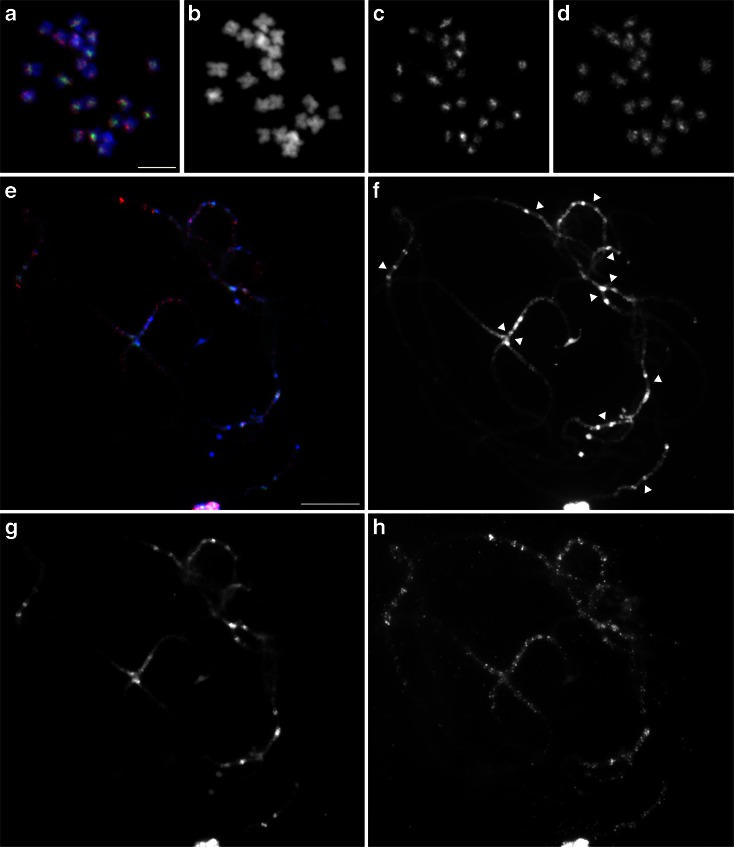
FISH images of pericentromeric BAC clones, VUH2_70J18 (*green*) and VUH2_81M23 (*red*), on mitotic metaphase (**a**–**d**) and meiotic pachytene chromosomes (**e**–**h**). **a**, **e** Merged images. **b**, **f** Chromosomes counterstained with DAPI. **c**, **g** 70J18 signals. **d**, **h** 81M23 signals. *Bars* = 5 μm (**a**) and 10 μm (**e**)

FISH of BACs, VUH2_70J18 (green) and VUH2_81M23 (red), on mitotic metaphase chromosomes showed that both BACs were distributed across pericentromeric regions of all chromosomes (Fig. 6a–d). Since we wanted to correlate BAC signals with chromosomal structure, pachytene chromosomes were used to further investigate the chromosomal localization of these BACs (Fig. 6e–h). FISH signals from both BAC were at the pericentromeres with distinct distribution patterns. VUH2_70J18 was primarily localized at the very condensed heterochromatic knob-like structures flanking the centromeres, whereas VUH2_81M23 was more dispersed along chromosome arms than VUH2_70J18. Signals of both BACs were very weak or not detected in centromeric regions (centromeres are shown with arrowheads in Fig. 4f), indicating that the BACs consist primarily of pericentromeric repetitive sequences. Part of the signals of VUH2_70J18 and VUH2_81M23 overlapped which indicates that both BAC clones may contain some common repeat sequences or interspersed with each other.

To better understand the genomic makeup of pericentromeric regions, we sequenced BAC VUH2_81M23 using Sanger sequencing technology. The BAC DNA was sequenced to phase II as there are four gaps in the BAC sequence, even though the coverage of this BAC is high, ∼21×. Seven genes were annotated (Supplemental Table 3), three of which were duplicated, and repetitive DNA was found throughout the sequence (Supplemental Fig. 3). Gene duplications interspersed with repetitive DNA were also found in another legume (Lin et al. 2010).

Two intact gypsy LTR retrotransposons were annotated, and their insertions truncate the 5′ ends of two previous LTR retrotransposon insertions. The intact LTR retrotransposon, located at 10,901–20,911 bp, is abundant in the cowpea genome based on BLASTN against a customized cowpea repeat database. Another region with multiple hits against the customized repeat database was located from 59,080 to 59,606 bp and belongs to repeat sequence, fam26-16498. Both the LTR retrotransposons and repeat sequence fam26-16498 have abundant hits from the analysis of 500-bp windows against the repeat database, indicating that both may contribute to the dispersed chromosomal distribution signal of this BAC across pericentromere regions. In several plant genomes, LTR retrotransposons and tandem repeats are known to be major components of pericentromeric regions (Jiang et al. 2002; Feng et al. 2002; Lin et al. 2005; Chang et al. 2008). We PCR amplified the LTR region of the intact LTR retrotransposon on 10,901–20,911 bp to examine its distribution. FISH signals of the LTR showed that it is concentrated in pericentromeric heterochromatic regions (Supplemental Fig. S4a, b), confirming that it is a major constituent of pericentromeric regions of cowpea.

The other pericentromeric BAC clone VUH2_70J18 was sequenced using 454 sequencing technology (Roche) resulting in 91,460 nonredundant reads. Since the read lengths were short (441 bp on average), it was not possible to assemble the entire sequence of the BAC. Instead, we conducted repeat analysis using RepeatExplorer which was developed to use 454 short reads as input (Novak et al. 2010, 2013). Similarity-based clustering of the short reads resulted in 50 clusters and included 90,738 reads. The top 18 clusters (more than 2816 reads per individual clusters) were further analyzed using RepeatMasker with the custom transposon database of soybean (Du et al. 2010) and common bean (http://www.phytozome.net/). Eighteen percent and nearly 50 % of the reads in the cluster 5 (CL5) showed hits against gypsy retrotransposons in soybean and common bean, respectively. We further analyzed the contigs assembled from the short reads contained within CL5 by the CAP3 program as implemented in RepeatExplorer. Two (CL5Contig3 and 7) out of 12 assembled contigs were 1.8 and 1.4 kb long, and a BlastX query against GenBank indicated the presence of gag-pol polyprotein in these contigs, further confirming the presence of a LTR retrotransposon in BAC VUH2_70J18. To confirm that the LTR retrotransposon is a genomic component of the knob-like heterochromatin in pericentromeres, the gag-pol region was amplified from the BAC DNA and used as a FISH probe which targeted the heterochromatic knob-like regions, confirming that this retrotransposon is a constituent of these unusual pericentromeric knobs (Supplemental Fig. 4c, d).

Next, we determined the presence of any common repeats in the two pericentromeric BACs, VUH2_70J18 and VUH2_81M23, using BLAST. Sequences from BAC VUH2_70J18 were used with BLASTN to query against assembled VUH2_81M23 sequence with a cutoff e-value 1E−4. Only one common repeat in both BACs, Gypsy LTR retrotransposon B, was found (Supplemental Fig. 3). Alignment of Gypsy LTR retrotransposon B sequences from the two BACs was short, 27, 71, and 75 bp. Thus, even though both BACs were physically located in pericentromeres, there were few shared sequences between the BACs suggesting a either a high rate of sequence change for the gypsy LTR retrotransposon or that it is an ancient element. These results are consistent with distinct distribution patterns of the two BACs within the pericentromere regions.

### Identification of a potential centromeric DNA

In cowpea, large, light-staining centromeres were observed on pachytene chromosomes which are intriguing given that plant centromeres typically consist of megabase-sized arrays of tandem repeats and retrotransposons (Jiang et al. 2003). Previously, a *DraI*-digested genomic DNA clone, pVuKB1, was reported to be distributed at all centromeric regions of cowpea (Galasso et al. 1995). However, the detailed structure of centromeric regions in cowpea was still undetermined. The most abundant tandem repeats in a plant genome are often centromeric (Gill et al. 2009; Melters et al. 2013). Tandem repeats finder (TRF, Benson 1999) has been used to identify potential centromeric or highly abundant tandem repeats from genome assemblies or sequences (Gill et al. 2009; Melters et al. 2013; Iwata et al. 2013). Therefore, we ran TRF against BESs derived from the VUH2 BAC library.

Since TRF results show only the copy numbers of tandem repeats within a BES, we used a set of 26-bp sequences that occurred frequently in a 60× cowpea draft genome to estimate the abundance of the tandem repeats in the whole genome. The set of 26-bp sequences were sorted according to frequency and used as a query for BLAST analysis against BES-derived tandem repeats

We found that a 176 bp (GenBank seq ID: EI903718.1) and a 445 bp (GenBank seq ID: EI938113.1) were the first and second most abundant tandem repeats in the genome. BLAST analysis revealed that part of the 176-bp tandem repeat was similar to the rDNA intergenic spacer subrepeat (Unfried et al. 1991) also confirmed by FISH showing co-localization of the 176-bp tandem repeat and 18S rDNA (Supplemental Fig. 5). We detected additional strong signals of the 176-bp tandem repeat (arrows on Supplemental Fig. 5c) at the proximal region of one pair of homologous chromosomes indicating independent amplification of the rDNA intergenic spacer subrepeat in cowpea.

**Fig. 7:**
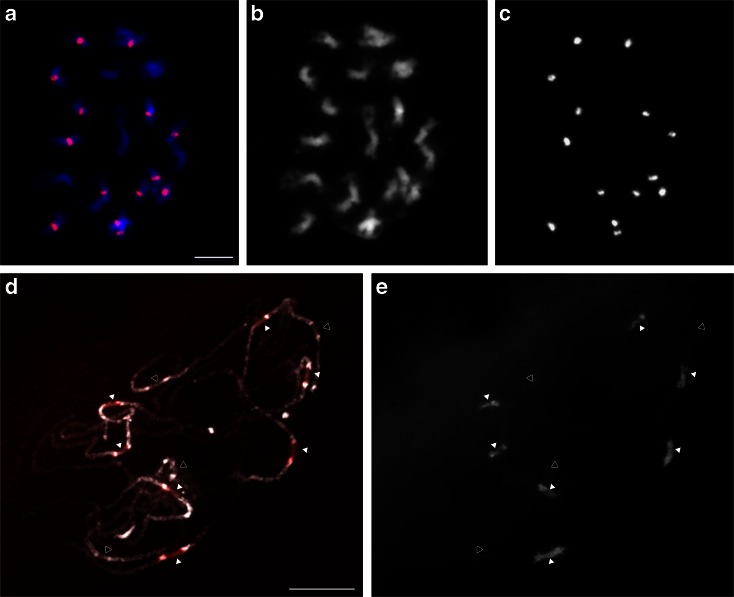
FISH analysis of potential centromeric repeats, 455-bp tandem repeat on mitotic prometaphase (**a**–**c**) and meiotic pachytene chromosomes (**d**, **e**). **b** Chromosomes counterstained with DAPI. **c**, **e** Signals of 455-bp tandem repeat. **a**, **d** Merged images. *Closed arrowheads* in **d**, **e** indicate centromeres with strong FISH signals, and *open arrowheads* indicate centromeres with very weak FISH signals. *Bars* = 5 μm (**a**) and 10 μm (**d**), respectively

Based on FISH, the 455-bp tandem repeat was found at seven of the 11 pairs of centromeres (chromosome 1, 2, 4, 5, 6, 10, and 11; closed arrowheads in Fig. 7). The 455-bp FISH signals covered the large centromeric regions of the seven centromeres and part of the flanking pericentromeric regions. Very weak FISH signals were also detected on the other four pairs of centromeres of pachytene chromosomes indicating the presence of very few or diverged sequences (open arrowheads in Fig. 7). None of these two tandem repeats showed sequence identity with pVuKB1; therefore, we used pVuKB1 sequence as a query to BLAST against the tandem repeats identified from the BESs and found a 285-bp (EI917305.1, EI917305.1, EI920835.1) tandem repeat with 80 % sequence identity to pVuKB1 (91st to 351st base). FISH using the 285-bp satellite repeat as a probe, however, showed distribution at pericentromeric regions of only one chromosome, possibly because the cowpea strain used in this study is different from the one used in Galasso et al. (1995), or our probe is missing bases 1 to 90 of the pVuKB1 clone (Supplemental Fig. 6). BLAST analysis using the first 90 bp of pVuKB1 sequence showed no hits against any identified tandem repeats of our TRF analysis. It is possible that the pVuKB1 sequence is organized independently from tandem repeats including the 285-bp tandem repeat. Another possibility is that the pVuKB1 sequence is not present at centromeres in accessions that we used as BLAST analysis using the first 90 bp of pVuKB1 sequence against BESs, and the genome assembly showed that they are present at relatively low copy numbers.

### Chromosomal distribution of cytosine DNA methylation in cowpea

**Fig. 8:**
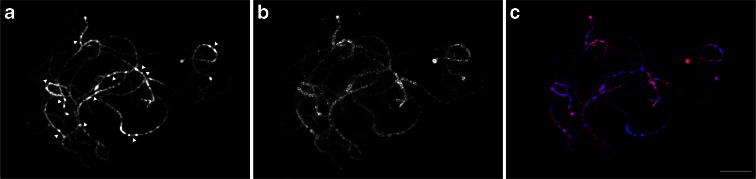
Immunodetection of 5-methylcytosine on pachytene chromosomes. **a** Chromosomes counterstained with DAPI. *Arrowheads* indicate centromeres. **b** Immunosignals of 5-methylcytosine. **c** Merged image of immunosignals (*red*) on chromosomes (*blue*). *Bar* = 10 μm

Since cowpea pachytene chromosomes have very distinct large centromeres flanked by unusually highly condensed heterochromatin, we wanted to explore the correlation between 5-methylcytosine distribution and these unusual cytological features. Immunostaining was performed on meiotic pachytene chromosomes using an antibody against 5-methylcytosine (Fig. 8). Signals were distributed across all the chromosomes, although more intense signals were observed in all pericentromeric regions. In contrast, centromeric regions had no or very weak 5-methylcytosine signals (arrowheads on Fig. 8a). Heterochromatic knobs at chromosomal termini, composed of 18S rDNA, and the one in the long arm of chromosome 4 showed very strong 5-methylcytosine signals.

## Discussion

Molecular cytogenetics has been useful to correlate genome sequences with chromosomal structures, which can lead to a more comprehensive understanding of the functional and structural properties of genomes. We used genomic resources and cytogenetics to advance our knowledge of cowpea chromosome and genome structure and to integrate the genetic and chromosomal maps.

Chromosome identification is the basis of much molecular cytogenetics work. Especially for species with small chromosomes (2–5 μm of mitotic metaphase chromosomes), it is nearly impossible to distinguish individual chromosomes using classical cytogenetics approaches, e.g., Giemsa and acetocarmine staining and length, arm ratios, and banding patterns. Pachytene chromosomes are much longer than mitotic metaphase chromosomes and provide higher resolution for analysis of cytological features (reviewed in de Jong et al. 1999; Jiang and Gill 2006). For example, cowpea pachytene chromosomes are >30 times longer than mitotic metaphase chromosomes. Therefore, we used pachytene chromosomes to provide more detailed description of chromosome structures of cowpea.

For several plants, chromosome-specific BAC clones have been used to identify individual chromosomes and integrate genetic and cytological maps (Dong et al. 2000; Cheng et al. 2001b; Chang et al. 2007; Koo et al. 2008; Tang et al. 2009; Fonseca et al. 2010; Xiong and Pires 2011). The 22 BAC clones used as a FISH probes in this study enabled us to integrate the genetic and chromosome maps and will accelerate cytogenetics studies in cowpea and other *Vigna* species that require chromosome identification. Vasconcelos et al. (2015) recently showed overall macrosynteny as well as several chromosomal rearrangements between common bean and cowpea based on cytogenetics mapping using common bean BAC clones. The tools developed here can be also used to investigate the conservation and evolution of chromosomal structures within *Vigna* species as well as to the closely related genus *Phaseolus*.

Genetic maps are mathematical representations of recombination and may not reflect actual physical distribution of recombination along a chromosome that can be biased due to nonrandom distribution of crossovers. Thus, FISH mapping on pachytene chromosomes using BAC clones containing genetic markers can reveal the distribution of recombination along a chromosome (Walling et al. 2006) which can provide insight into recombination hot/cold spots and the coverage of genetic map along individual chromosomes. In this study, we mapped genetically anchored BAC clones located near the ends of linkage groups to subtelomeric regions of chromosomes, which indicates that those linkage groups span the entire chromosomes.

Variation of recombination frequency can be associated with local chromosomal structures. Typically, recombination frequencies decrease at heterochromatic pericentromeres and centromeres, while increasing at distal ends of chromosomes in species such as maize, wheat, barley, sorghum, tomato, and rice (Gill et al. 1996; Wang et al. 2006a; Kim et al. 2005; Künzel et al. 2000; Szinay et al. 2008; Zhao et al. 2002). In addition to pericentromeres, recombination frequencies might be attenuated in two other chromosomal regions of cowpea accession used in this study such as the following: (1) unpaired termini of chromosomes 4, 6, and 10 and (2) the heterochromatic knob on the long arm of chromosome 4. The short arms of chromosome 4, 6, and 10 were often unpaired at pachytene, the stage where homologous chromosome synapse and recombination occur. We do not think that this is a cytological artifact, as it was consistently observed and only for a few chromosomes. It would be interesting to see if the unpaired termini are found in other cowpea strains or are specific to IT97K-499-35. Since IT97K-499-35 is a homozygous strain, based on SNP analysis (personal communication with Dr. Timothy Close, University of California, Riverside), we do not expect that this is due to structural heterozygosity. Further experiment, such as immunofluorescence with elements of synaptonemal complex such as meiotic asynaptic mutant 1 protein (ASY1) and zipper 1-like protein (ZYP1) (Armstrong et al. 2002; Higgins et al. 2005), may help in our understanding of this phenomena.

We found at least two different Ty3 gypsy LTR retrotransposons occupying the pericentromeres of cowpea that have very different distribution patterns—one highly condensed into knob-like structures and the other dispersed throughout the pericentromeres. Repetitive DNA sequences constitute a large fraction of plant genomes and contribute to variation in genome size and content among related species. Pericentromeric heterochromatic regions in plant genomes are composed primarily of highly repetitive sequences, including retrotransposons and tandem repeats (Jiang et al. 2002; Feng et al. 2002; Lin et al. 2005; Wang et al. 2006b; Chang et al. 2008). While the structure of pachytene chromosomes in cowpea resembles other plants with similar genome sizes, what was unique was the presence of very conspicuous heterochromatic knob-like structures flanking all centromeres. Based on cytogenetics and sequence analysis of BAC clone VUH2_70J18 that specifically hybridized to the knob-like structures, we confirmed that retrotransposons are major constituents of these structures. Given that pachytene chromosomes of common bean do not have this highly condensed knob-like structures in pericentromeres (unpublished data), we hypothesize that this retrotransposon inserted and then rapidly amplified to form these highly distinct heterochromatic regions in cowpea pericentromeres after divergence from common bean (∼5 MYA). What the functional significance of these structures might be is still unclear.

Heterochromatic knobs at chromosomal termini and/or in euchromatic regions have been observed in maize, *Arabidopsis*, rice, *Antirrhinum majus*, common bean, tomato, and several crucifer species plant genomes (Fransz et al. 2000; Cheng et al. 2001b; Lamb et al. 2007; Zhang et al. 2008; Chang et al. 2008; Mandakova and Lysak 2008; David et al. 2009). In these species, the heterochromatic knobs are composed of either rDNA or tandem repeats. In cowpea, there were heterochromatic knobs at chromosomal termini composed of rDNA and an interstitial knob at the short arm of chromosome 4. The genomic component of the interstitial knob is still unknown, but the knob likely arose after the divergence from common bean based on the observation of common bean pachytene chromosomes (David et al. 2009; Fonseca et al. 2014; our unpublished data). In common bean, there are small heterochromatic knobs at most chromosomal termini composed of the *khipu* tandem repeat (David et al. 2009). Consistent with Southern analysis and FISH experiment (David et al. 2009; Vasconcelos et al. 2015), we did not observe any *khipu*-related heterochromatic knobs at chromosomal termini of cowpea. Given that common bean and cowpea have high levels of synteny (http://harvest.ucr.edu/; Vasconcelos et al. 2015), repetitive sequences must have evolved rapidly to form species-specific sequences and chromosome structures after the divergence of common bean and cowpea ∼5 MYA.

In plants, centromeres typically consist of megabase-sized arrays of satellite repeats and centromere-specific retrotransposons (reviewed in Jiang et al. 2003). On pachytene chromosomes, centromeres can be visualized after DAPI staining as condensed or decondensed structures depending on the species and even individual chromosomes (Cheng et al. 2001b; Chang et al. 2008). Observation of DAPI-stained pachytene chromosomes of cowpea showed that all 11 centromeric regions were highly decondensed, but not appearing like “typical” euchromatin or heterochromatin, and much larger than typical plant centromeres. It will be interesting to investigate if the centromeric histone H3, which is a mark of functional centromeres, uniformly occupies the entire centromeric regions or partially resides in the regions in “beads on a string” pattern such as seen in *Pisum sativum* (Neumann et al. 2012; 2015).

From our repeat analysis, we found a 455-bp tandem repeat that was highly abundant in the genome and mainly localized at seven of the 11 pairs of centromeres. The other four centromeres showed very weak FISH signals, indicating that the 455-bp tandem repeat is not a major component of these centromeres. We do not know what sequences underlie the other four centromere pairs as we did not uncover any other obvious high-copy tandem repeats except a 176-bp tandem repeat related to the rDNA intergenic spacer subrepeat. Major component of these four pairs of centromeres might consist of nonrepetitive sequences or nontandem repeats such as transposons. It is also possible that the pVuKB1 sequence, which is present at centromeres of other cowpea accessions, might be present at these centromeres. One hypothesis for centromere evolution is that centromeres evolved from “neocentromeres” that originally formed from single- or low-copy sequences to satellite repeat-based centromeres via invasion and fixation of the repeats at centromeres. This hypothesis is strongly supported in potato where there are six satellite repeat-based centromeres and five repeat-free centromeres (Gong et al. 2012). The presence of the 455-bp tandem repeat at a subset of centromeres indicates that this tandem repeat may still be in the process of fixation by accumulating at nonrepetitive centromeres or by competing with other repetitive sequences to dominate all centromeres.

High levels of cytosine DNA methylation (5-methylcytosine) have been found at repeat-rich heterochromatic pericentromeres in plants (Zhang et al. 2006; Fransz et al. 2006; Yan et al. 2010). Hypermethylation was seen in cowpea pericentromeres and the heterochromatic knob-like structures at chromosomal termini and chromosome 4. In contrast, hypomethylation was observed in cowpea centromeres. Hypomethylation of the centromeric DNA was also found in maize and *Arabidopsis*, despite the fact that centromeres are composed primarily of mega-sized arrays of tandem repeats and retrotransposons which are typically methylated (Zhang et al. 2008; Koo and Jiang 2009). In rice, hypermethylation and hypomethylation of centromeric DNA were observed and appeared to depend on the DNA composition at individual functional centromeres (Yan et al. 2010). Nucleosomes by virtue of their position determine accessibility to DNA methyltransferases and play a prominent role in determining the methylation pattern of a genome (Chodavarapu et al. 2010). The differences in nucleosome positioning/structure can either facilitate or prevent methylation of the associated DNA and thus may contribute to differences in the methylation patterns of centromeric and pericentromeric chromatin of cowpea. In addition to DNA methylation, histone modification patterns in centromeric chromatin are different from both euchromatin and flanking heterochromatin in human and *Drosophila melanogaster* (Sullivan and Karpen 2004), which may explain the distinct appearance of DAPI-stained centromeric chromatin.

This study provides foundational knowledge of cowpea chromosome structure and molecular cytogenetics tools for further chromosome studies in *Vigna* species. Cowpea has interesting chromosomal structures that merit further investigation, including the dense heterochromatic knobs flanking the highly de-condensed and large centromeric regions. The information provided here should aid in the ongoing genome sequencing project by understanding chromosome structure and the distribution of a few of the major repeat families.

## Electronic supplementary material

Below is the link to the electronic supplementary material.

Supplemental Figure 1 FISH images of 18S (green) and 5S (red) rDNAs on meiotic pachytene chromosomes. Bar = 10 μm. In addition to four major 18S rDNA loci that overlapped with large heterochromatic knobs at chromosomal termini, one strong signal was detected in pericentromeric heterochromatin (arrow) and one weak signal was detected at a small heterochromatic knob on chromosome 9S (arrowhead). (GIF 24 kb) (GIF 71 kb)

High resolution (TIF 21659 kb)

Supplemental Figure 2 FISH image showing the position of BAC clone, M051D16 on chromosome 7. The BAC clone is anchored to short arm of linkage group 7 (3.3 cM), but physically mapped onto the long arm of chromosome 7. (A) Merged image of chromosome (blue) and the BAC signal (red). Arrowhead indicates centromere of chromosome 7. (B) DAPI image. Bar = 10 μm. (GIF 24 kb)

High resolution (TIF 15251 kb)

Supplemental Figure 3 Sequence analysis of BAC VUH2_81M23. Top panel, a 500-bp non-overlapping sliding window (x-axis) was used to map the number of BLASTN hits (y-axis) from a search against the cowpea repeat database (generated by RECON analysis of VUH2 BES database) along the length of VUH2_81M23. Annotation of VUH2_81M23 is shown at bottom. Yellow pentagons represent genes 1- 7, and genes 1, 4, and 6 are duplicates shown with asterisks. Two light green boxes are intact LTR retrotransposons A and B (Gypsy). Dark green boxes are four truncated LTR retrotransposons a-d. Pink box shows the position of a repeat sequence, fam26-16498, with multiple hits against the customized repeat database. (PPTX 80 kb)

Supplemental Figure 4 FISH analysis of repetitive fraction of pericentromeric BAC clones on pachytene chromosomes counterstained with DAPI (blue). (A and B) FISH image of LTR region in VUH2_81M23 (red) (C and D). FISH image of gag-pol region in VUH2_70J18 (red). Bar = 10 μm. (GIF 112 kb)

High resolution (TIF 51753 kb)

Supplemental Figure 5 FISH analysis of 176-bp tandem repeat (green) and 18S rDNA (red) on mitotic metaphase chromosomes. (C) Signals of 176-bp tandem repeat. Arrows indicate independent amplification of 176-bp tandem repeat from ribosomal genes. (D) Signals of 18S rDNA. Bar = 5 μm. (GIF 39 kb)

High resolution (TIF 16664 kb)

Supplemental Figure 6 FISH image of 285-bp tandem repeat (green) on pachytene chromosomes (Arrow). 285-bp tandem repeat signal overlaps with pericentromeric heterochromatin. Bar = 10 μm. (GIF 45 kb)

High resolution (TIF 14162 kb)

ESM 1 (PDF 13 kb)

ESM 2 (PDF 10 kb)

ESM 3 (PDF 9 kb)

ESM 4 (PPTX 241 kb)

## Abbreviations

ASY1: Meiotic asynaptic mutant 1 protein
BAC: Bacterial artificial chromosome
BESs: Bacterial artificial chromosome end sequences
CDS: Coding segments
CL: Cluster
DAPI: 4′,6-Diamidino-2-phenylindole
FISH: Fluorescence in situ hybridization
MYA: Million years ago
rDNA: Ribosomal DNA
TRF: Tandem repeat finder
ZYP1: Zipper 1-like protein
