# Supplementary figures and images for: Highly distinct chromosomal structures in cowpea (Vigna unguiculata), as revealed by molecular cytogenetic analysis
Source: Chromosome Res. 2016 Jan 12;24:197–216. doi: 10.1007/s10577-015-9515-3 (PMC4856725; doi:10.1007/s10577-015-9515-3)

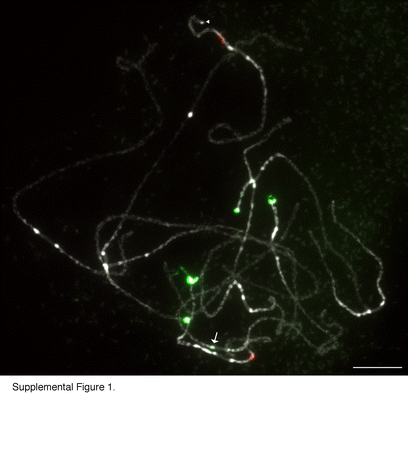

Supplement: Supplementary file 1 — FISH images of 18S (green) and 5S (red) rDNAs on meiotic pachytene chromosomes. Bar = 10 μm. In addition to four major 18S rDNA loci that overlapped with large heterochromatic knobs at chromosomal termini, one strong signal was detected in pericentromeric heterochromatin (arrow) and one weak signal was detected at a small heterochromatic knob on chromosome 9S (arrowhead). (GIF 24 kb) (GIF 71 kb) [file 10577_2015_9515_Fig9_ESM.gif]

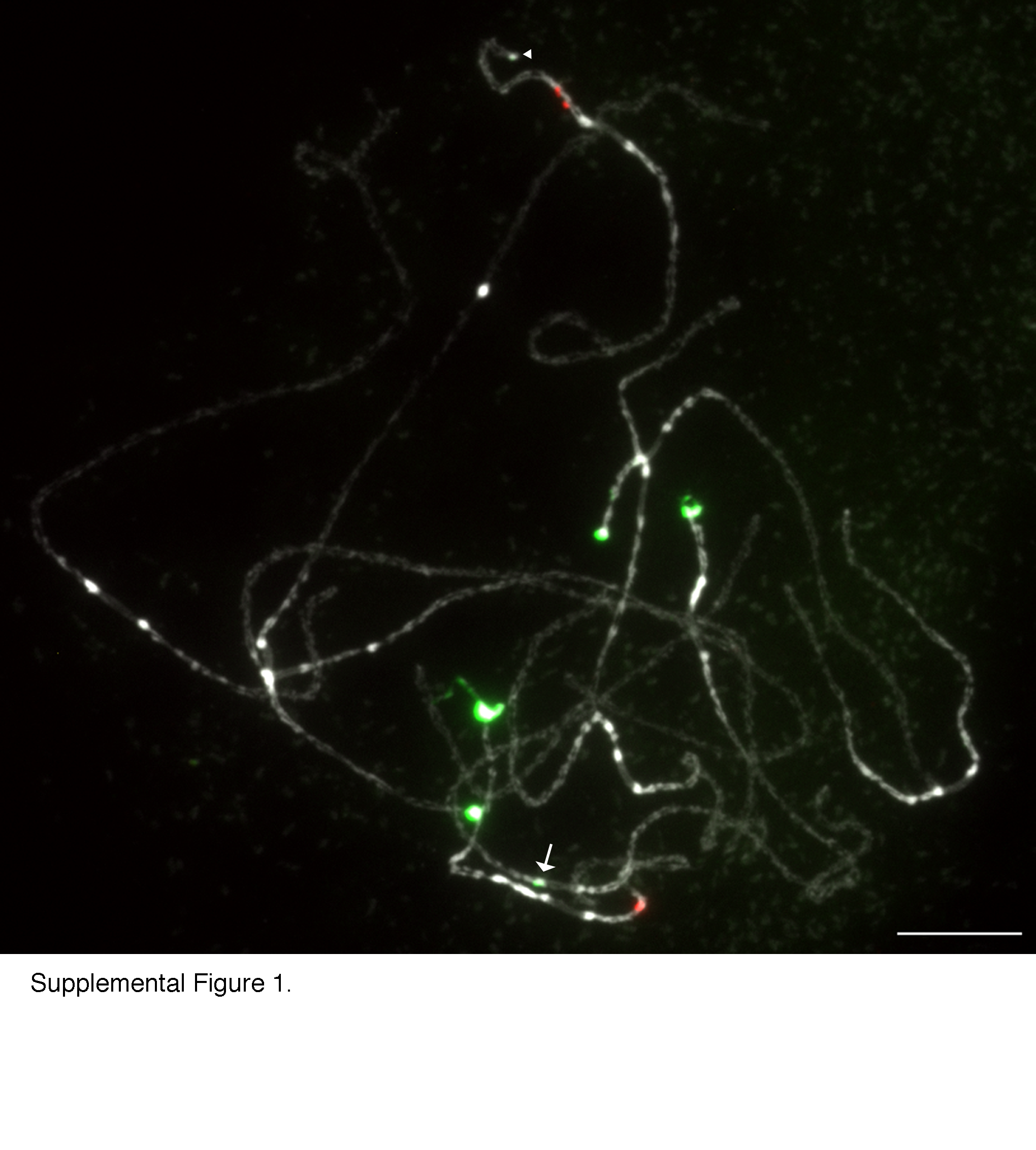

Supplement: Supplementary file 2 — High resolution (TIF 21659 kb) [file 10577_2015_9515_MOESM1_ESM.tif]

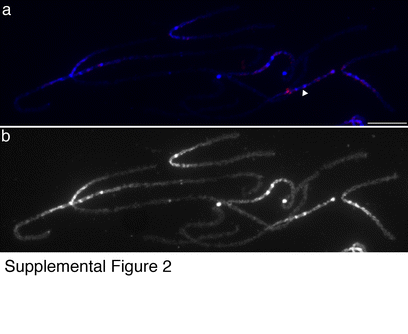

Supplement: Supplementary file 3 — FISH image showing the position of BAC clone, M051D16 on chromosome 7. The BAC clone is anchored to short arm of linkage group 7 (3.3 cM), but physically mapped onto the long arm of chromosome 7. (A) Merged image of chromosome (blue) and the BAC signal (red). Arrowhead indicates centromere of chromosome 7. (B) DAPI image. Bar = 10 μm. (GIF 24 kb) [file 10577_2015_9515_Fig10_ESM.gif]

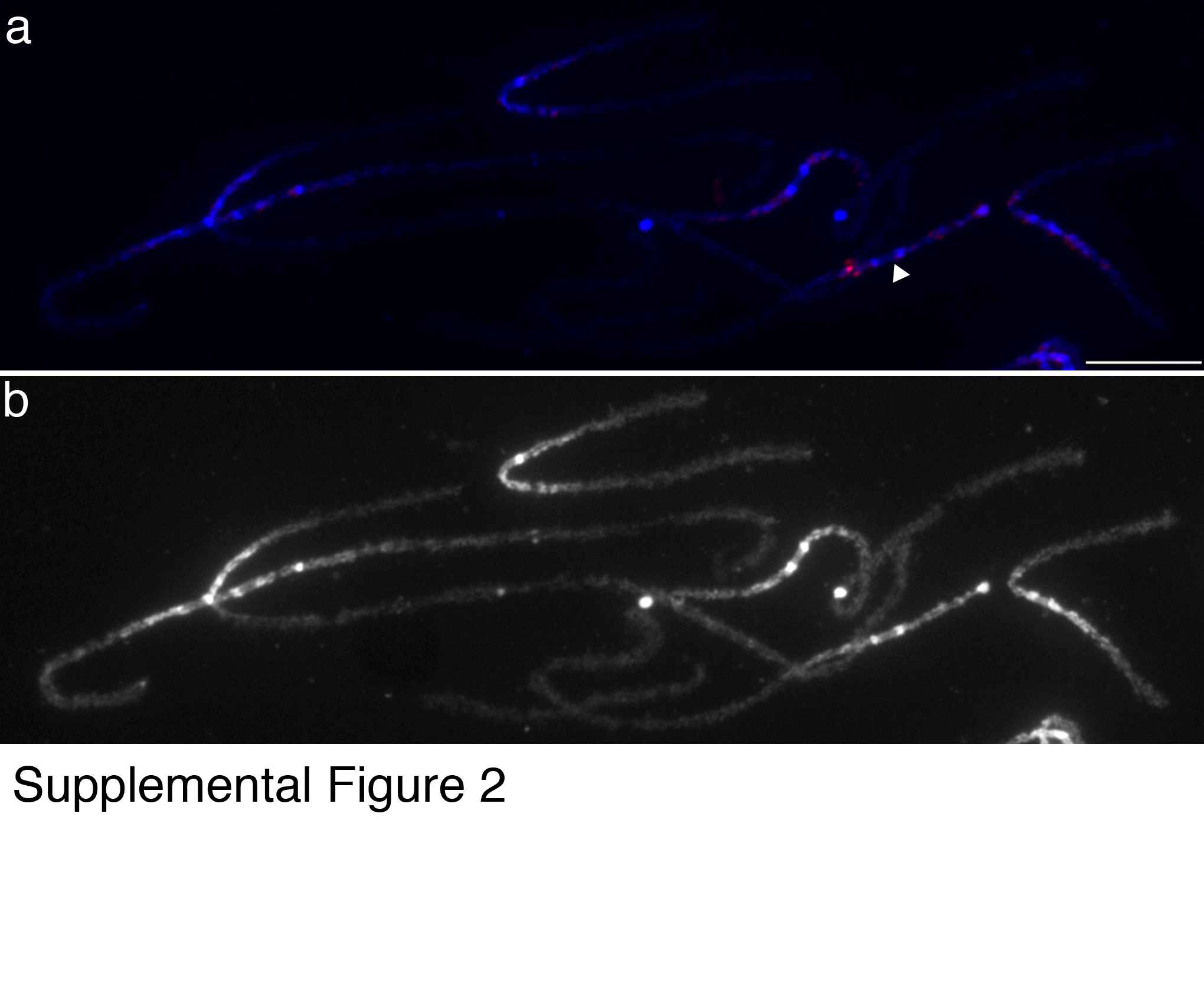

Supplement: Supplementary file 4 — High resolution (TIF 15251 kb) [file 10577_2015_9515_MOESM2_ESM.tif]

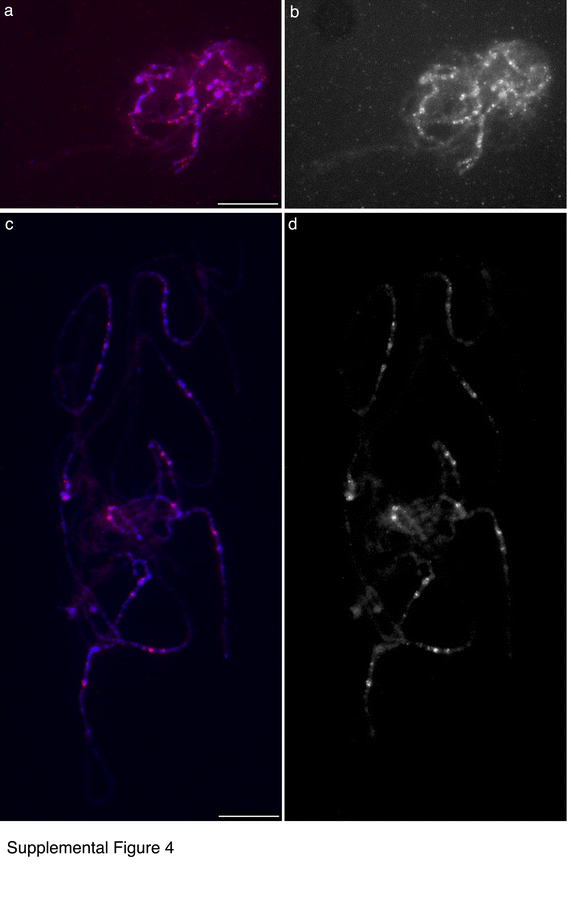

Supplement: Supplementary file 6 — FISH analysis of repetitive fraction of pericentromeric BAC clones on pachytene chromosomes counterstained with DAPI (blue). (A and B) FISH image of LTR region in VUH2_81M23 (red) (C and D). FISH image of gag-pol region in VUH2_70J18 (red). Bar = 10 μm. (GIF 112 kb) [file 10577_2015_9515_Fig11_ESM.gif]

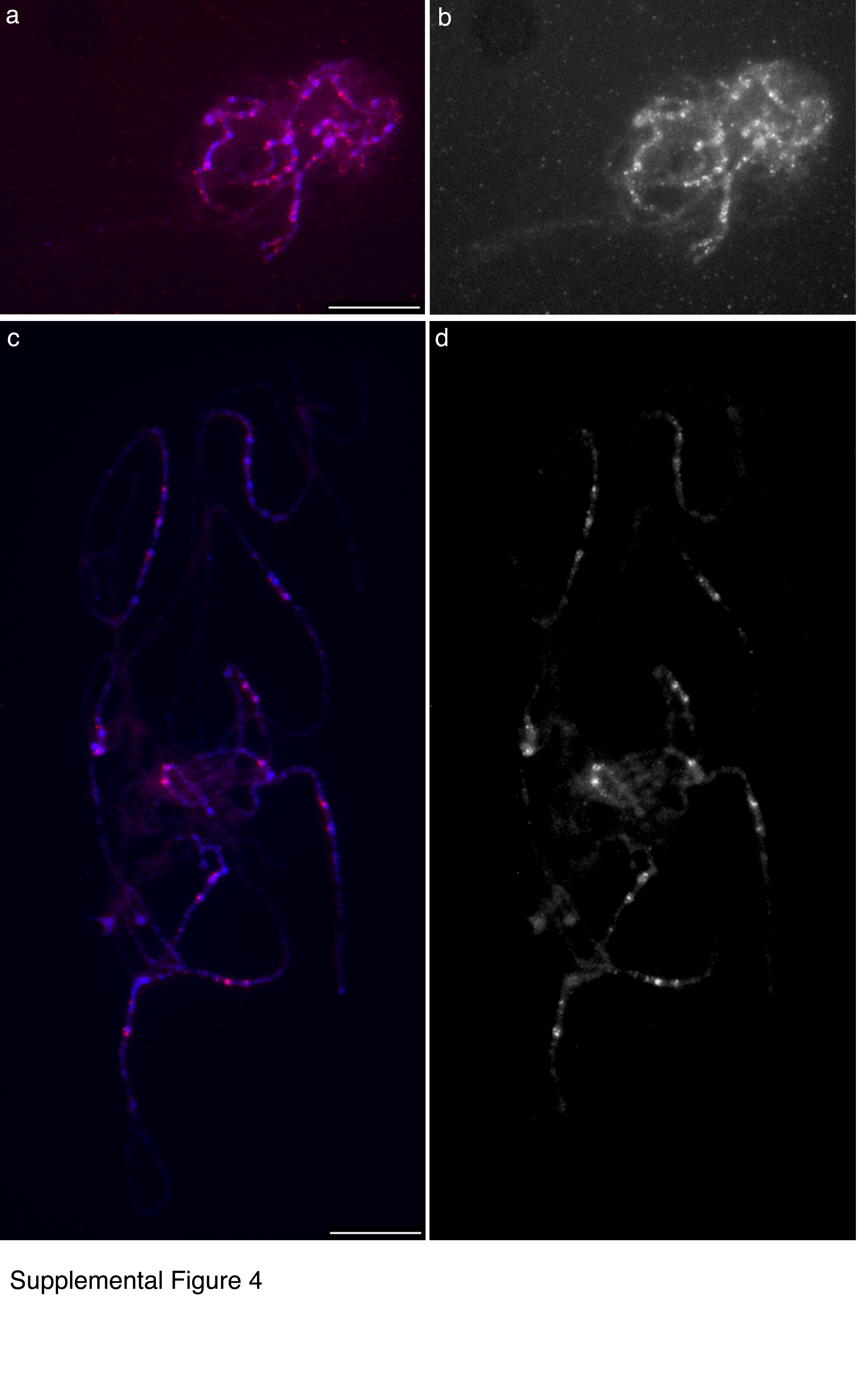

Supplement: Supplementary file 7 — High resolution (TIF 51753 kb) [file 10577_2015_9515_MOESM4_ESM.tif]

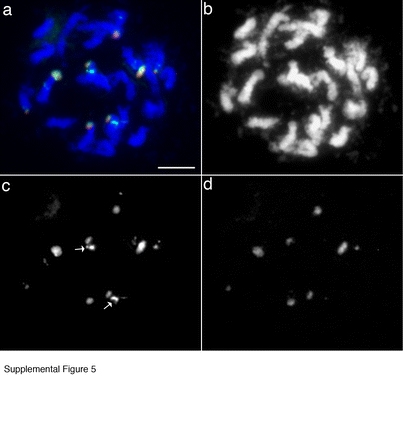

Supplement: Supplementary file 8 — FISH analysis of 176-bp tandem repeat (green) and 18S rDNA (red) on mitotic metaphase chromosomes. (C) Signals of 176-bp tandem repeat. Arrows indicate independent amplification of 176-bp tandem repeat from ribosomal genes. (D) Signals of 18S rDNA. Bar = 5 μm. (GIF 39 kb) [file 10577_2015_9515_Fig12_ESM.gif]

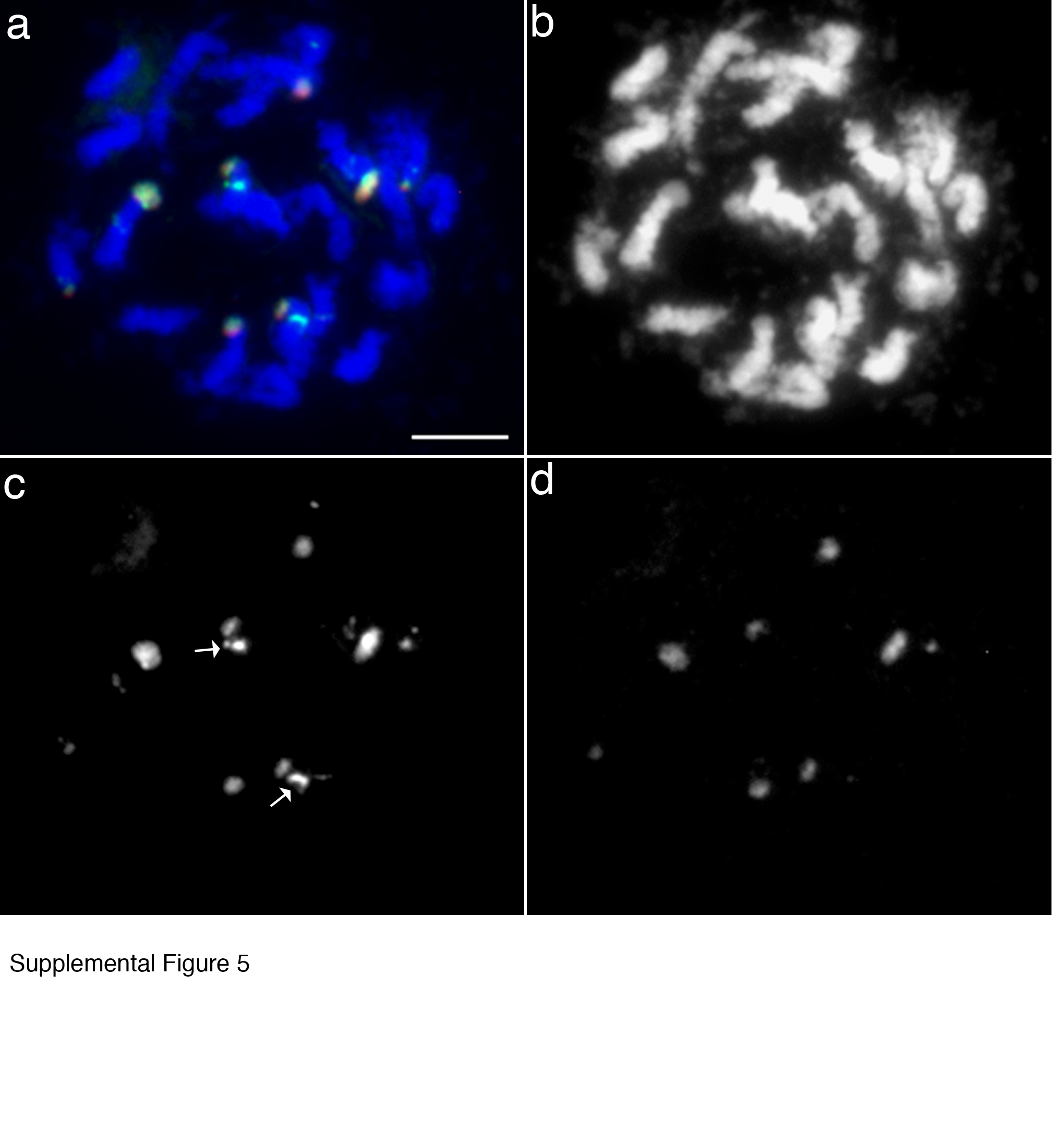

Supplement: Supplementary file 9 — High resolution (TIF 16664 kb) [file 10577_2015_9515_MOESM5_ESM.tif]

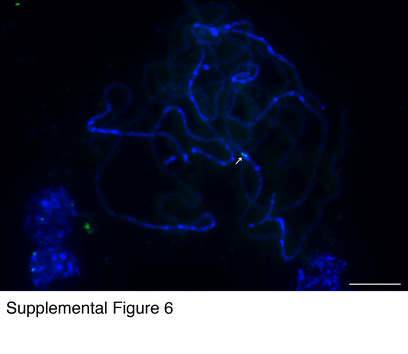

Supplement: Supplementary file 10 — FISH image of 285-bp tandem repeat (green) on pachytene chromosomes (Arrow). 285-bp tandem repeat signal overlaps with pericentromeric heterochromatin. Bar = 10 μm. (GIF 45 kb) [file 10577_2015_9515_Fig13_ESM.gif]

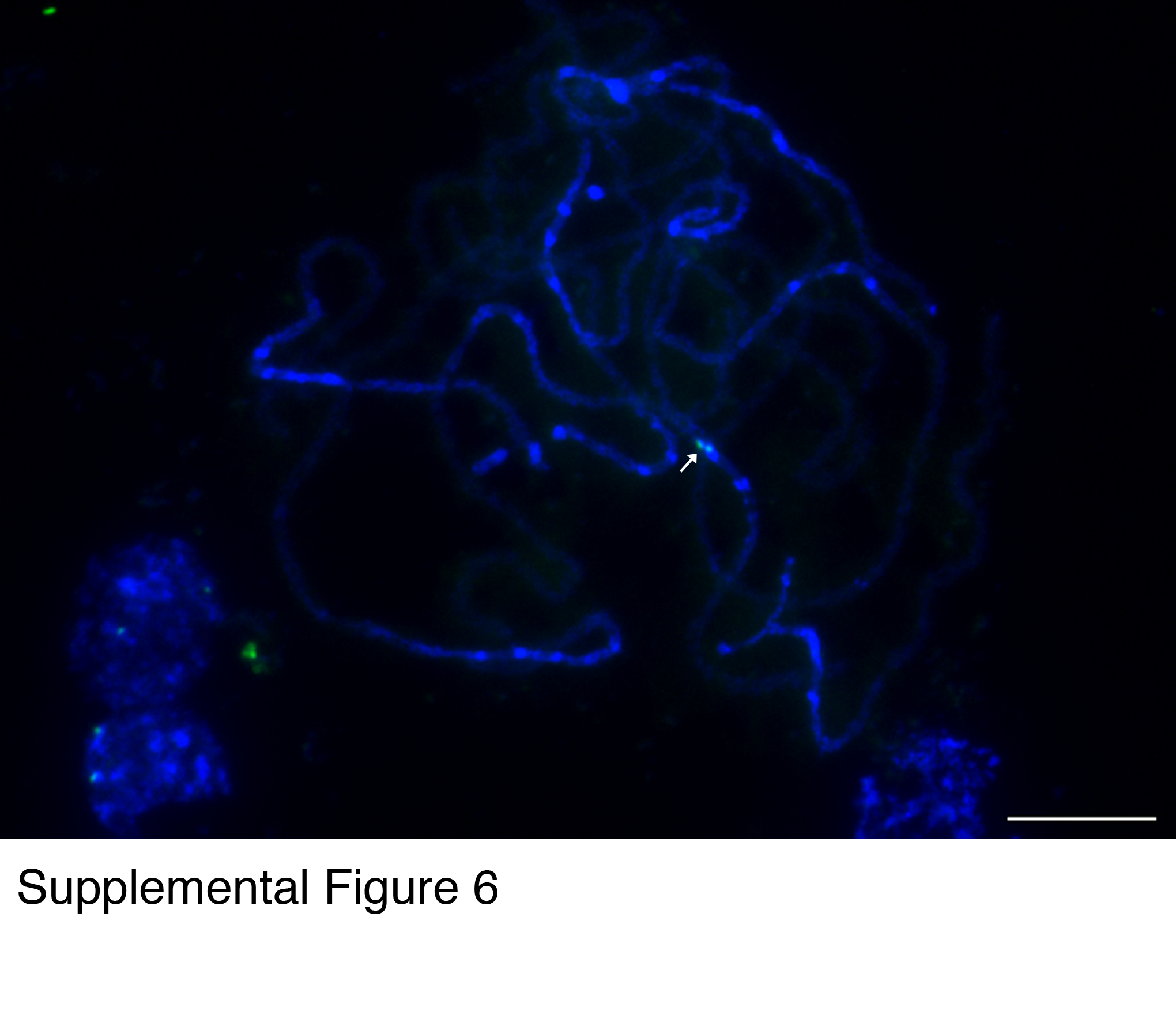

Supplement: Supplementary file 11 — High resolution (TIF 14162 kb) [file 10577_2015_9515_MOESM6_ESM.tif]
