# Supplementary material for: Highly distinct chromosomal structures in cowpea (Vigna unguiculata), as revealed by molecular cytogenetic analysis
Source: Chromosome Res. 2016 Jan 12;24:197–216. doi: 10.1007/s10577-015-9515-3 (PMC4856725; doi:10.1007/s10577-015-9515-3)
Supplement: Supplementary file 5 — Sequence analysis of BAC VUH2_81M23. Top panel, a 500-bp non-overlapping sliding window (x-axis) was used to map the number of BLASTN hits (y-axis) from a search against the cowpea repeat database (generated by RECON analysis of VUH2 BES database) along the length of VUH2_81M23. Annotation of VUH2_81M23 is shown at bottom. Yellow pentagons represent genes 1- 7, and genes 1, 4, and 6 are duplicates shown with asterisks. Two light green boxes are intact LTR retrotransposons A and B (Gypsy). Dark green boxes are four truncated LTR retrotransposons a-d. Pink box shows the position of a repeat sequence, fam26-16498, with multiple hits against the customized repeat database. (PPTX 80 kb) [file 10577_2015_9515_MOESM3_ESM.pptx]

## Slide 1
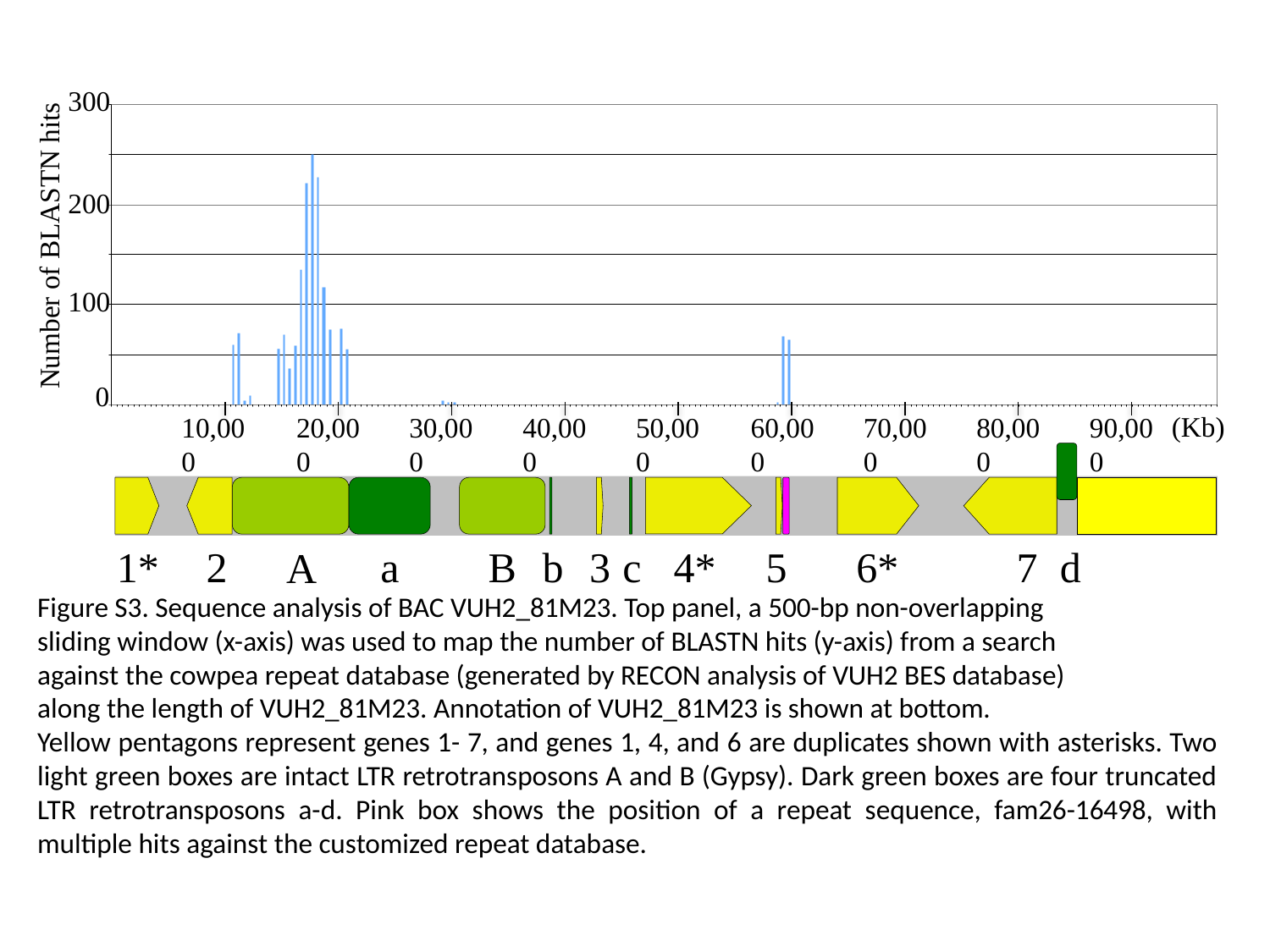

300
200
100
0
Number of BLASTN hits
 (Kb)
10,000
20,000
30,000
40,000
50,000
60,000
70,000
80,000
90,000
1*
2
a
b
3
c
4*
5
6*
7
d
B
A
Figure S3. Sequence analysis of BAC VUH2_81M23. Top panel, a 500-bp non-overlapping
sliding window (x-axis) was used to map the number of BLASTN hits (y-axis) from a search
against the cowpea repeat database (generated by RECON analysis of VUH2 BES database)
along the length of VUH2_81M23. Annotation of VUH2_81M23 is shown at bottom.
Yellow pentagons represent genes 1- 7, and genes 1, 4, and 6 are duplicates shown with asterisks. Two light green boxes are intact LTR retrotransposons A and B (Gypsy). Dark green boxes are four truncated LTR retrotransposons a-d. Pink box shows the position of a repeat sequence, fam26-16498, with multiple hits against the customized repeat database.

## Slide 2
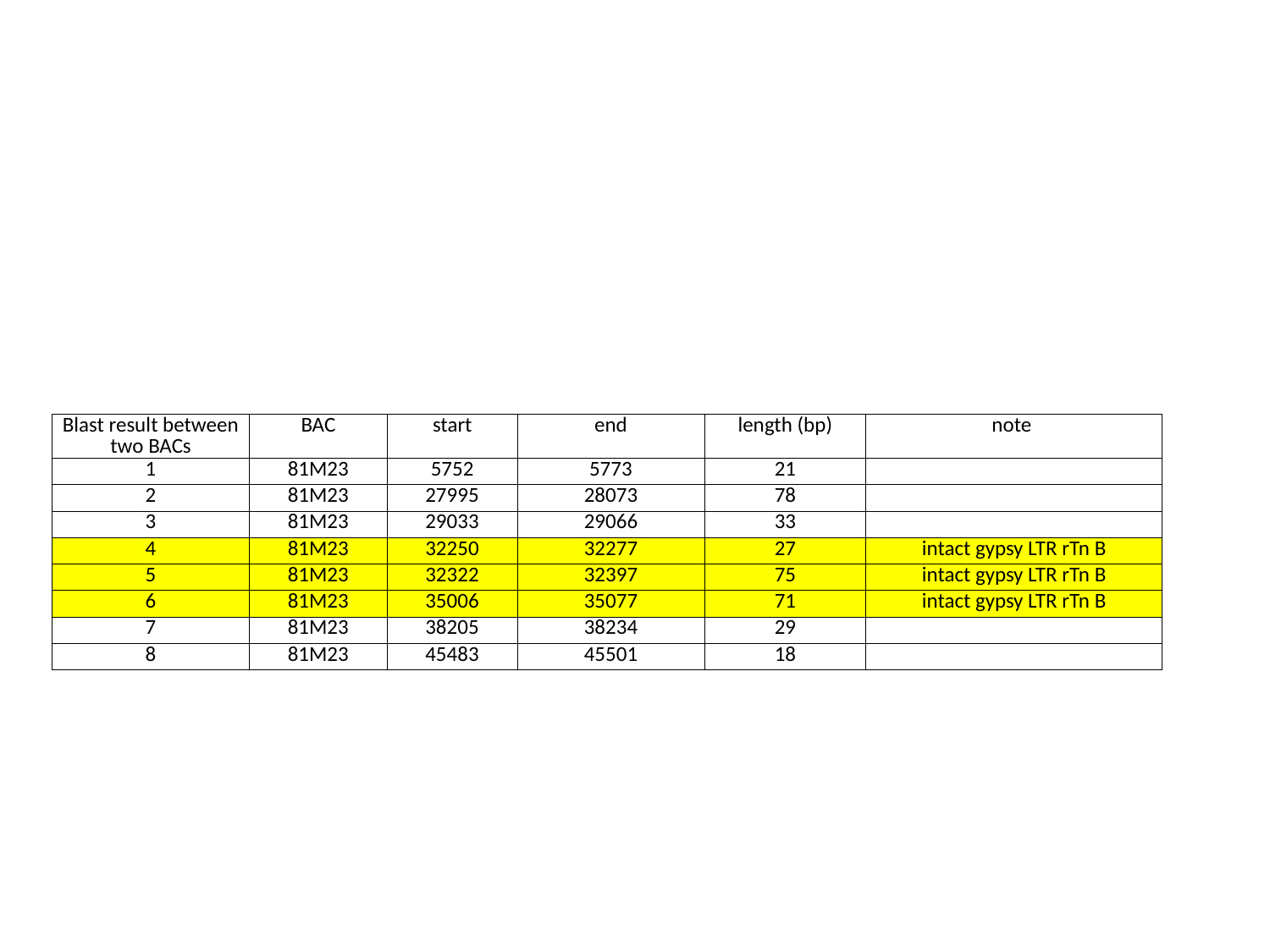

#
| Blast result between two BACs | BAC | start | end | length (bp) | note |
| --- | --- | --- | --- | --- | --- |
| 1 | 81M23 | 5752 | 5773 | 21 | |
| 2 | 81M23 | 27995 | 28073 | 78 | |
| 3 | 81M23 | 29033 | 29066 | 33 | |
| 4 | 81M23 | 32250 | 32277 | 27 | intact gypsy LTR rTn B |
| 5 | 81M23 | 32322 | 32397 | 75 | intact gypsy LTR rTn B |
| 6 | 81M23 | 35006 | 35077 | 71 | intact gypsy LTR rTn B |
| 7 | 81M23 | 38205 | 38234 | 29 | |
| 8 | 81M23 | 45483 | 45501 | 18 | |
