# Supplementary material for: Highly distinct chromosomal structures in cowpea (Vigna unguiculata), as revealed by molecular cytogenetic analysis
Source: Chromosome Res. 2016 Jan 12;24:197–216. doi: 10.1007/s10577-015-9515-3 (PMC4856725; doi:10.1007/s10577-015-9515-3)
Supplement: Supplementary file 12 — (PDF 13 kb) [file 10577_2015_9515_MOESM7_ESM.pdf]

Supplemental Table 1 Description of primers used in this study

Amplified sequence	Name of primer set	Template	Forward Primer Sequence (5’-3’)	Reverse Primer Sequence (5’-3’)
455-bp tandem repeat	P1	Genomic DNA of IT97k-499-35	GGATCAACATAGGCTG AAGGAG	TCATTTCATGTCCCT CATGG
285-bp tandem repeat	P2	Genomic DNA of IT97k-499-35	AGGAGATAAAAGAA(G) T(A)ACGCATGA	GATTGATTTCCACTG (A)CT(A)TTTTCA
gag-pol region of VUH2_70J18	P3	BAC DNA of VUH2_70J18	ACAATGACTTGGTGGC CTCA	GGCCATGGTTCTTT TGGTAGA
LTR region of VUH2_ 81M23	P4	BAC DNA of VUH2_81M23	GCCCACTATCTCTTCA GTTG	TGCTAGCCTACCAA CACATA
