# Supplementary material for: Highly distinct chromosomal structures in cowpea (Vigna unguiculata), as revealed by molecular cytogenetic analysis
Source: Chromosome Res. 2016 Jan 12;24:197–216. doi: 10.1007/s10577-015-9515-3 (PMC4856725; doi:10.1007/s10577-015-9515-3)
Supplement: Supplementary file 13 — (PDF 10 kb) [file 10577_2015_9515_MOESM8_ESM.pdf]

Supplemental Table 2 Frequencies (%) of unpaired chromosomal termini on four chromosomes (n=50).

	Unpaired	Partially paired	Fully paired
Chromosome 4	30	10	60
Chromosome 6	76	22	2
Chromosome 7	0	8	92
Chromosome 10	38	62	0
