# Supplementary material for: Highly distinct chromosomal structures in cowpea (Vigna unguiculata), as revealed by molecular cytogenetic analysis
Source: Chromosome Res. 2016 Jan 12;24:197–216. doi: 10.1007/s10577-015-9515-3 (PMC4856725; doi:10.1007/s10577-015-9515-3)
Supplement: Supplementary file 14 — (PDF 9 kb) [file 10577_2015_9515_MOESM9_ESM.pdf]

Supplemental Table 3 Annotation of BAC VUH2_81M23

Gene	Strand	Name
1	+	peptidyl-prolyl cis-trans isomerase cyclophilin-type family protein
2	-	TNP1
3	+	collagen-like protein
4	+	peptidyl-prolyl cis-trans isomerase cyclophilin-type family protein
5	+	expressed protein
6	+	peptidyl-prolyl cis-trans isomerase cyclophilin-type family protein
7	-	Kinesin heavy chain
