# Supplementary material for: Highly distinct chromosomal structures in cowpea (Vigna unguiculata), as revealed by molecular cytogenetic analysis
Source: Chromosome Res. 2016 Jan 12;24:197–216. doi: 10.1007/s10577-015-9515-3 (PMC4856725; doi:10.1007/s10577-015-9515-3)
Supplement: Supplementary file 15 — (PPTX 241 kb) [file 10577_2015_9515_MOESM10_ESM.pptx]

## Slide 1
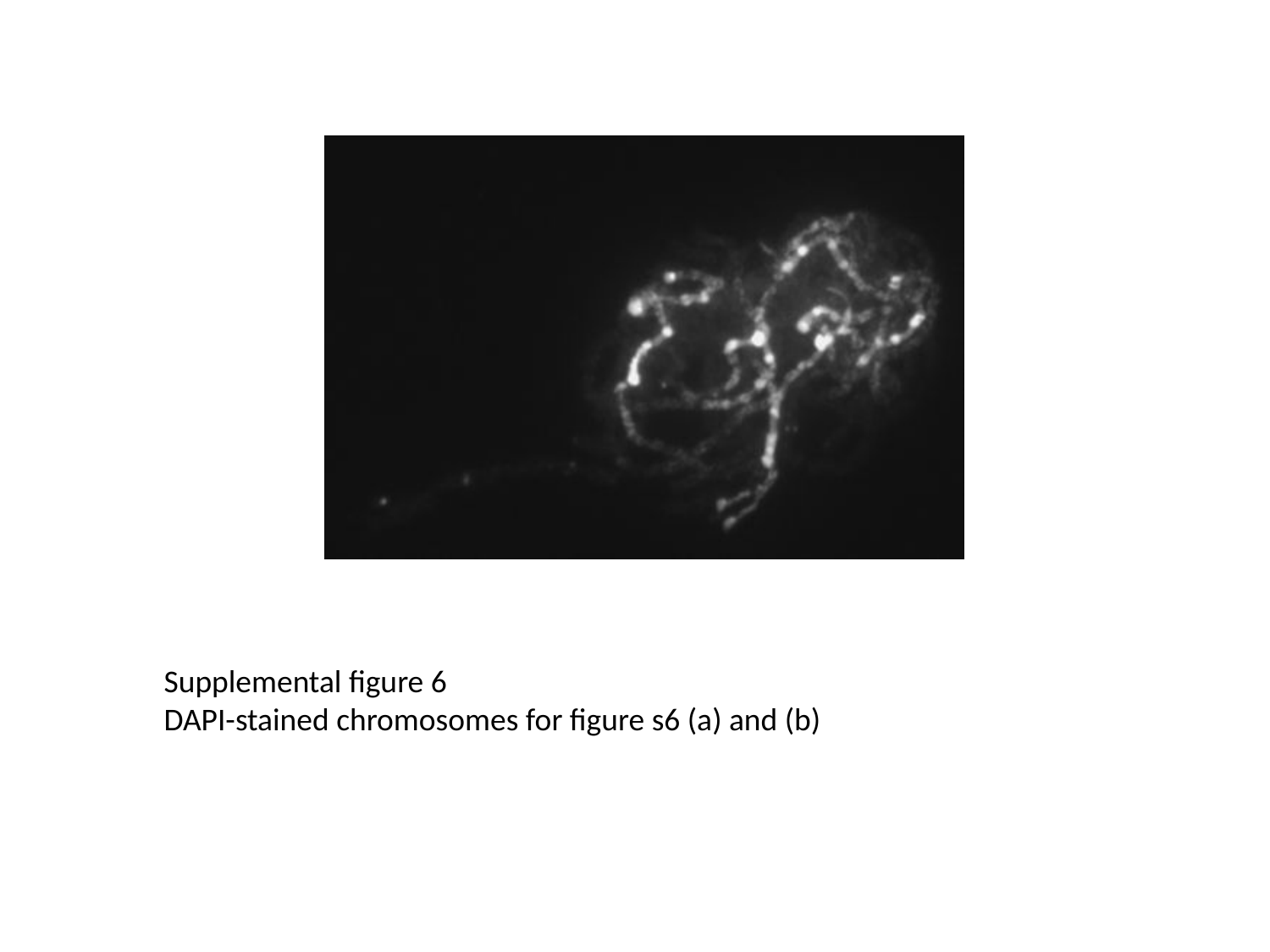

Supplemental figure 6
DAPI-stained chromosomes for figure s6 (a) and (b)

## Slide 2
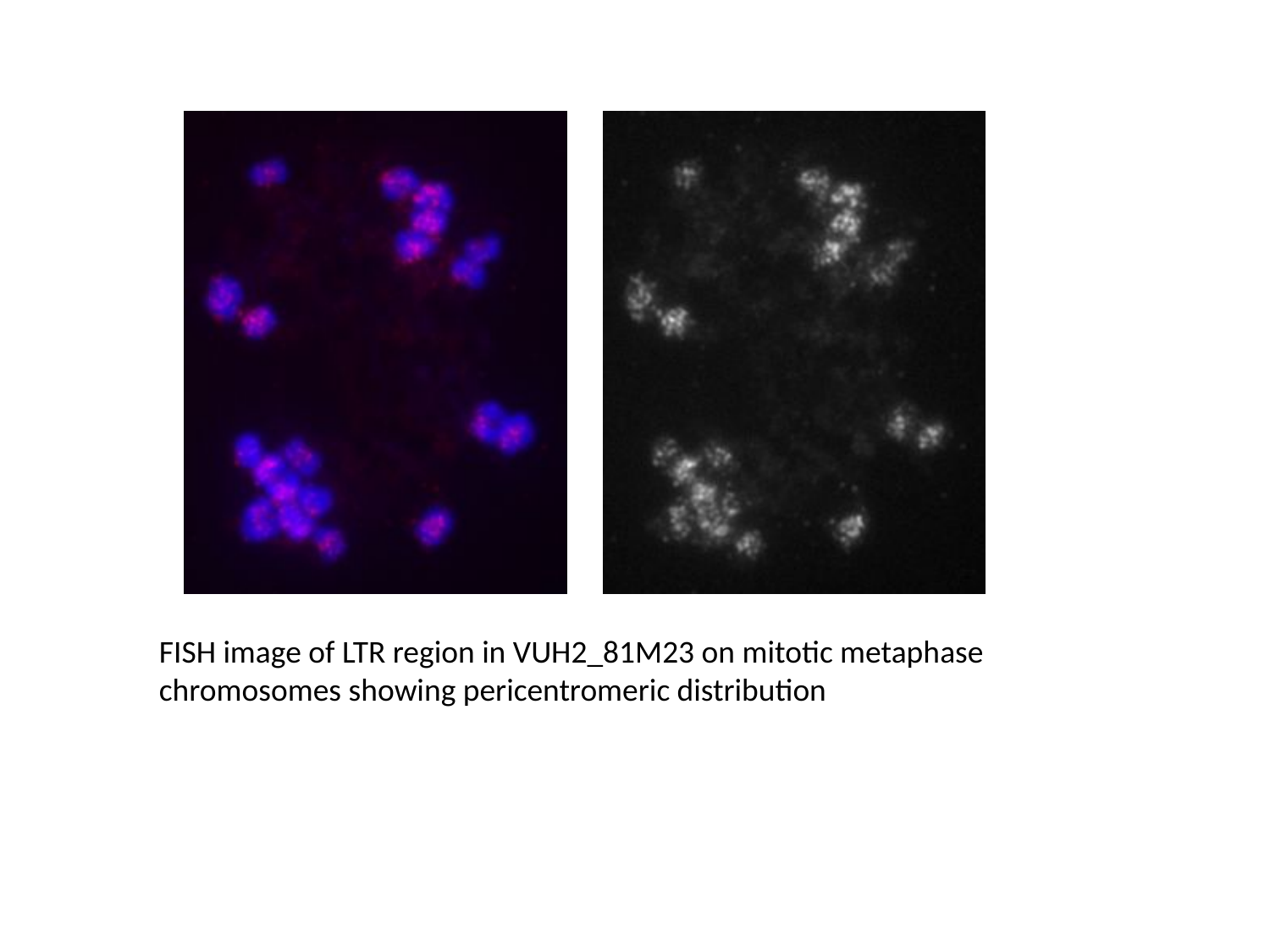

FISH image of LTR region in VUH2_81M23 on mitotic metaphase chromosomes showing pericentromeric distribution
